# Supplementary material for: Robust Segmentation of Lung Proton and Hyperpolarized Gas MRI with Vision Transformers and CNNs: A Comparative Analysis of Performance Under Artificial Noise
Source: Bioengineering (Basel). 2025 Jul 28;12(8):808. doi: 10.3390/bioengineering12080808 (PMC12383719; doi:10.3390/bioengineering12080808)
Supplement: Supplementary file 1 [file bioengineering-12-00808-s001.zip › bioengineering-3767663-supplementary.pdf]

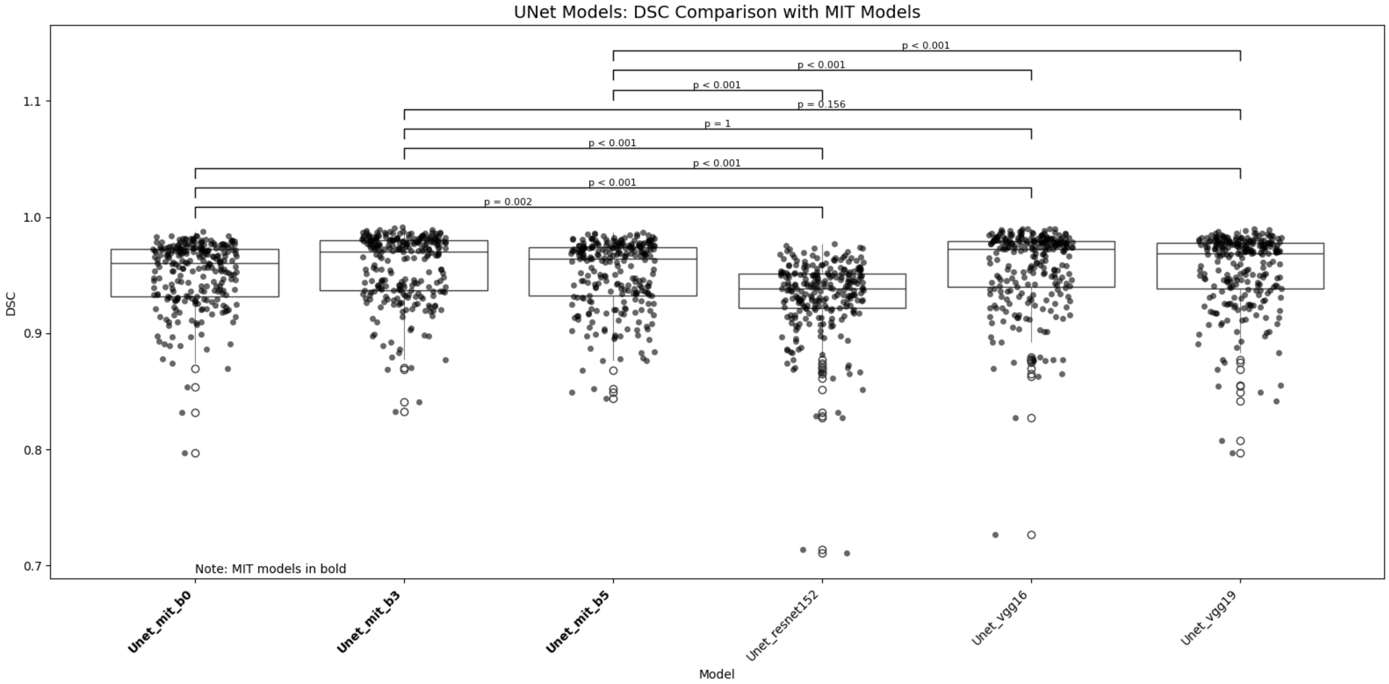

Figure S1. Boxplot of Dice Similarity Coefficient (DSC) distributions for UNet models under no-noise condition for Proton MRI. Under optimal imaging conditions, all models demonstrate strong segmentation performance with minimal differences between CNN and ViT-based backbones. The ViT-based models (MiT variants) show slightly tighter distributions, indicating more consistent performance across the dataset.

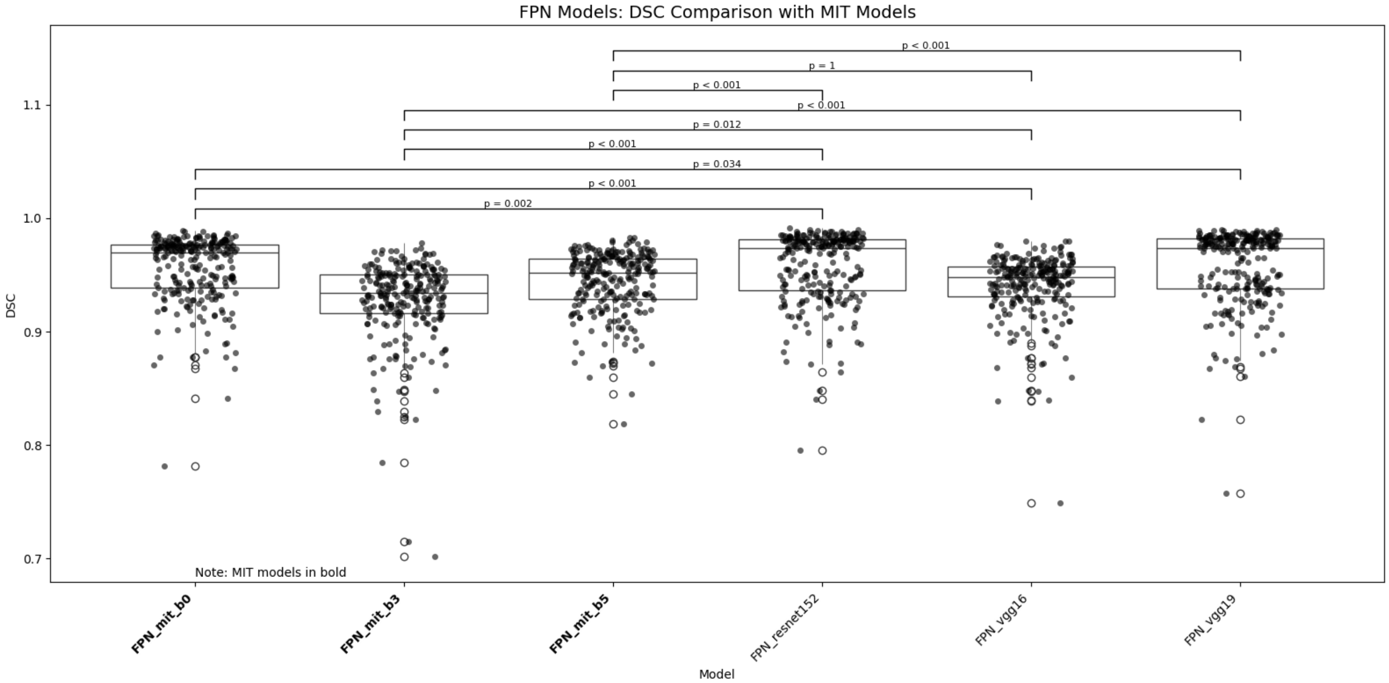

Figure S2. Boxplot of Dice Similarity Coefficient (DSC) distributions for FPN models under no-noise condition for Proton MRI. Similar to UNet architectures, FPN models achieve high DSC values in noise-free conditions, with MiT-based backbones demonstrating marginally superior performance and reduced variability compared to traditional CNN backbones.

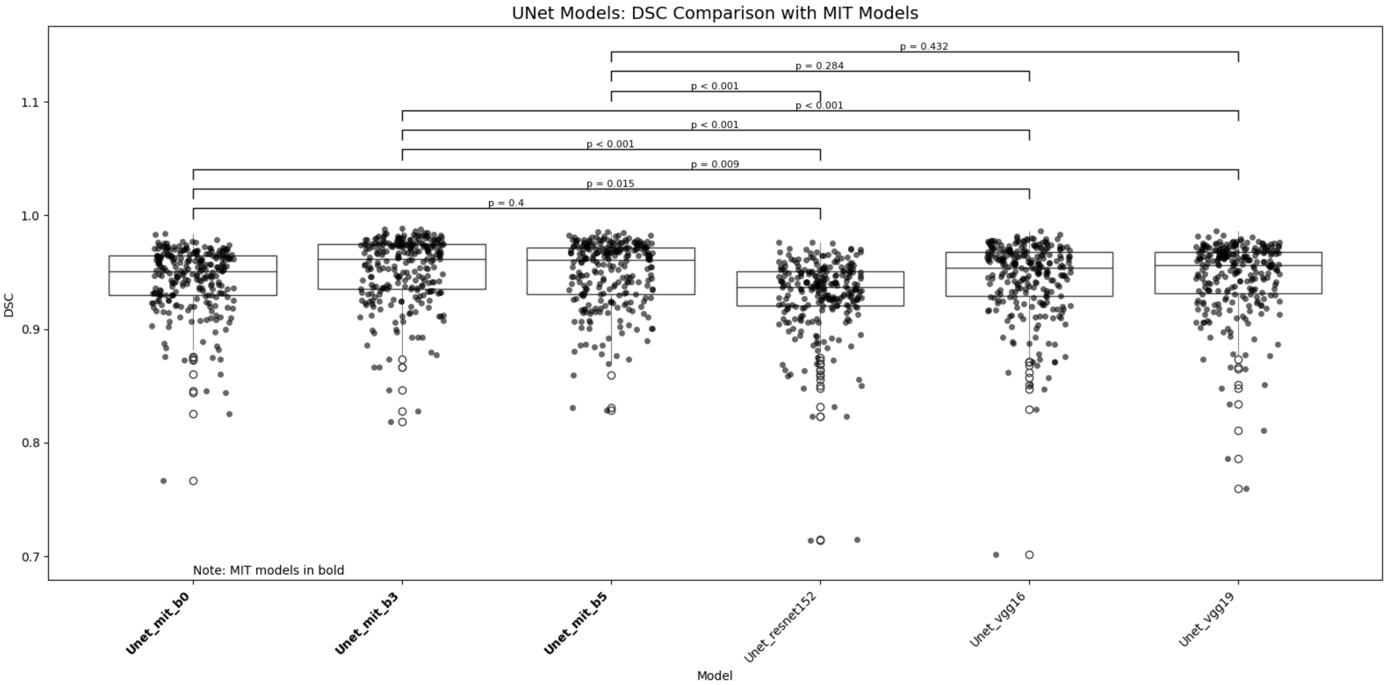

Figure S3. Boxplot of Dice Similarity Coefficient (DSC) distributions for UNet models under low-noise condition for Proton MRI. Introduction of low-level Gaussian noise (std = 0.05) begins to reveal performance differences, with ViT-based models maintaining higher median DSC values and tighter interquartile ranges. Statistical analysis confirmed significant differences between model groups ($p < 0.001$).

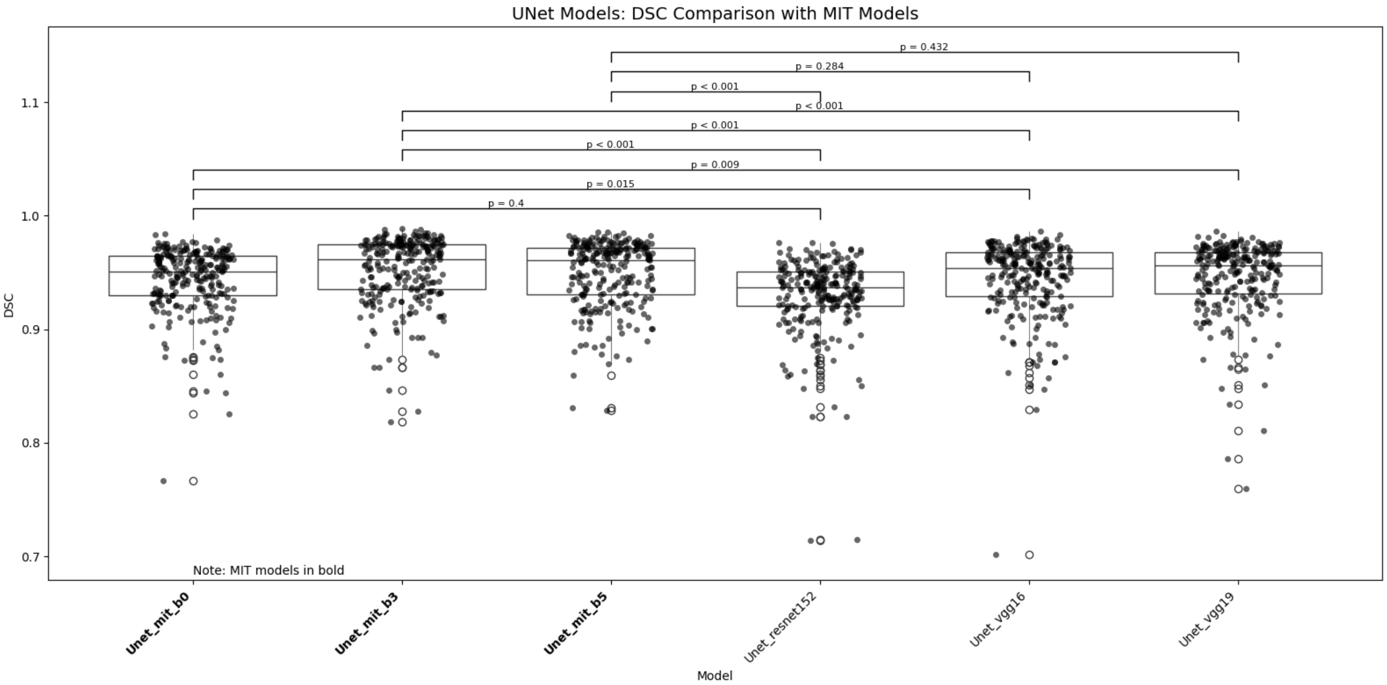

Figure S4. Boxplot of Dice Similarity Coefficient (DSC) distributions for FPN models under low-noise condition for Proton MRI. FPN architectures with MiT backbones demonstrate greater resilience to noise compared to CNN-based variants,

with notably fewer outliers and more stable performance distributions across the test dataset.

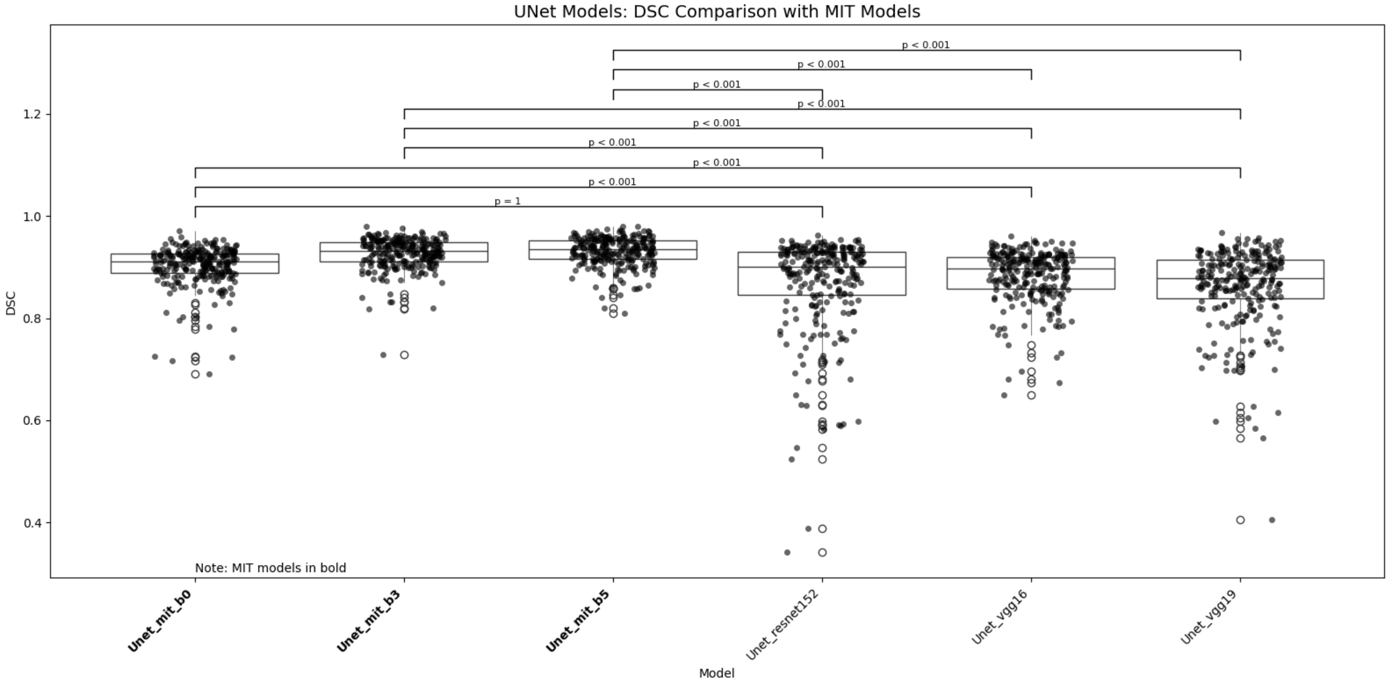

Figure S5. Boxplot of Dice Similarity Coefficient (DSC) distributions for UNet models under medium-noise condition for Proton MRI. At medium noise levels (std = 0.15), the performance gap between ViT and CNN models widens significantly. CNN-based models show increased variability and lower median performance, while transformer-based models maintain relatively stable segmentation accuracy.

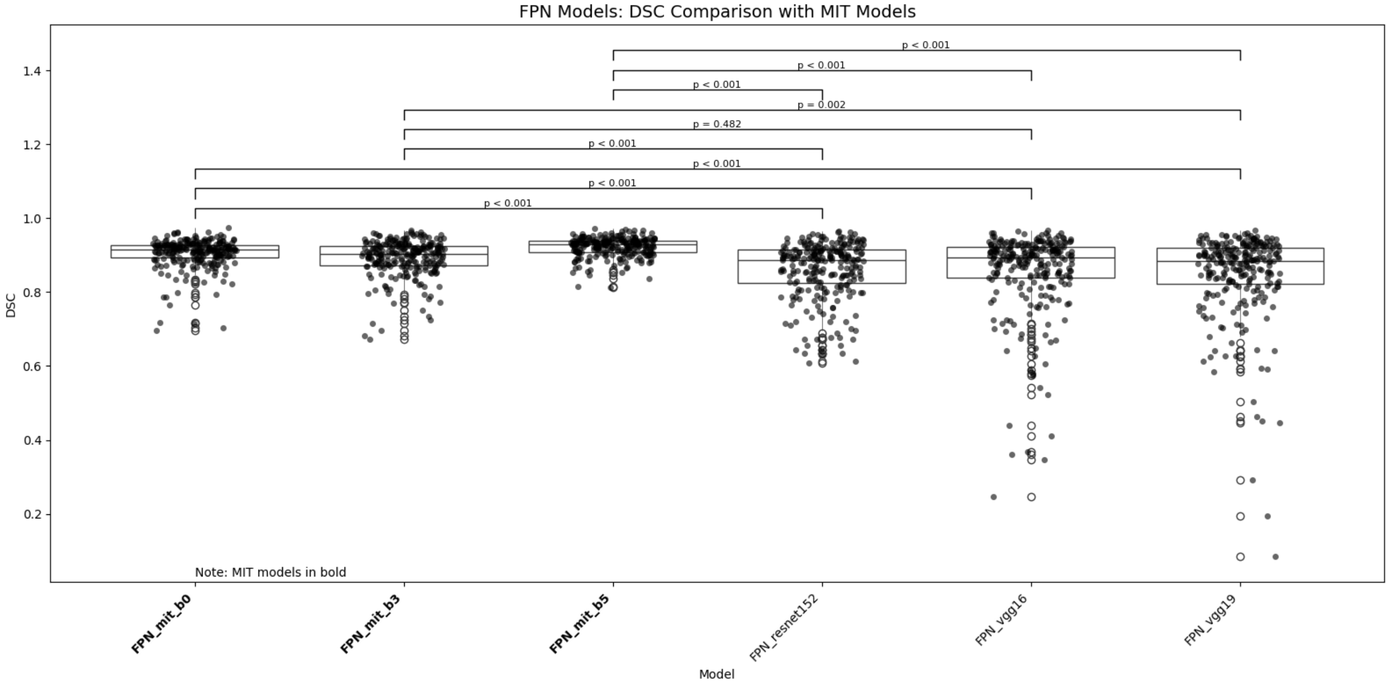

Figure S6. Boxplot of Dice Similarity Coefficient (DSC) distributions for FPN models under medium-noise condition for Proton MRI. The superiority of ViT-based FPN models becomes increasingly evident under medium noise conditions, with MiT backbones achieving significantly higher DSC scores and demonstrating superior robustness to image degradation.

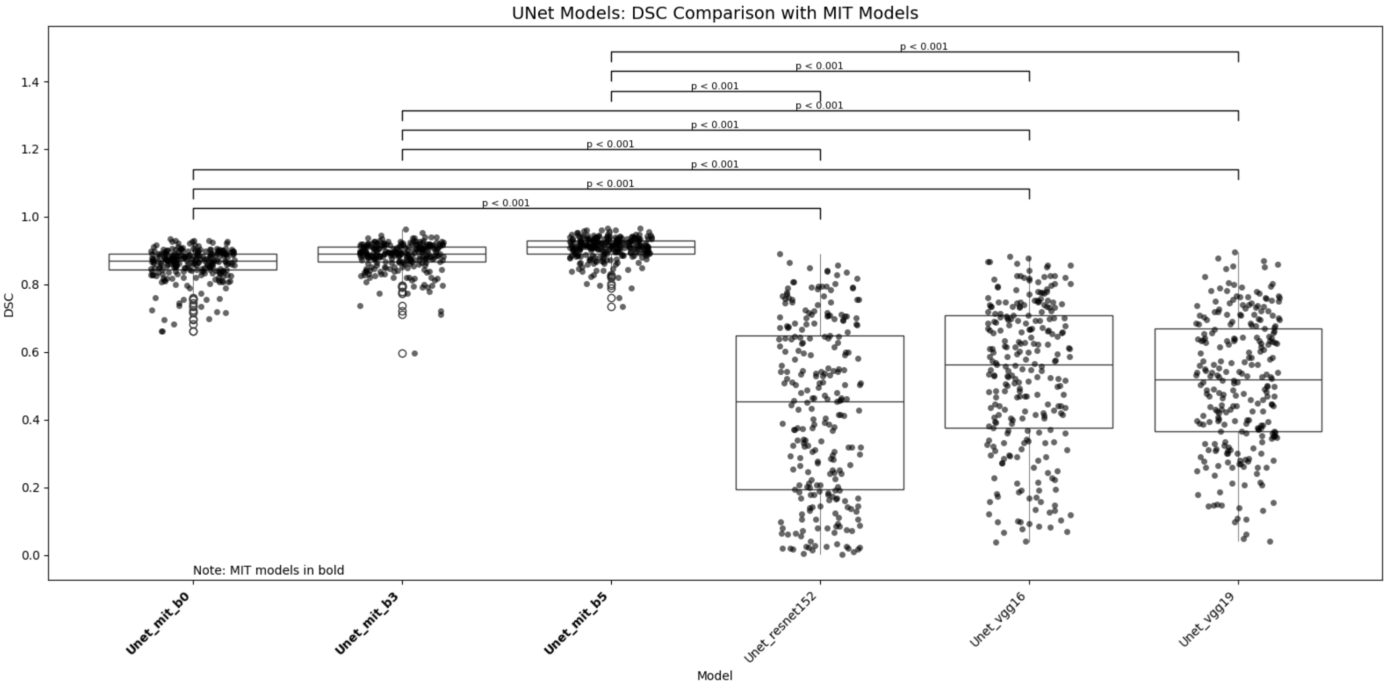

Figure S7. Boxplot of Dice Similarity Coefficient (DSC) distributions for UNet models under high-noise condition for Proton MRI. Under challenging high-noise conditions (std = 0.25), CNN-based models suffer substantial performance degradation with wide distributions and numerous outliers. In contrast, ViT-based models maintain acceptable segmentation quality, highlighting their superior noise tolerance.

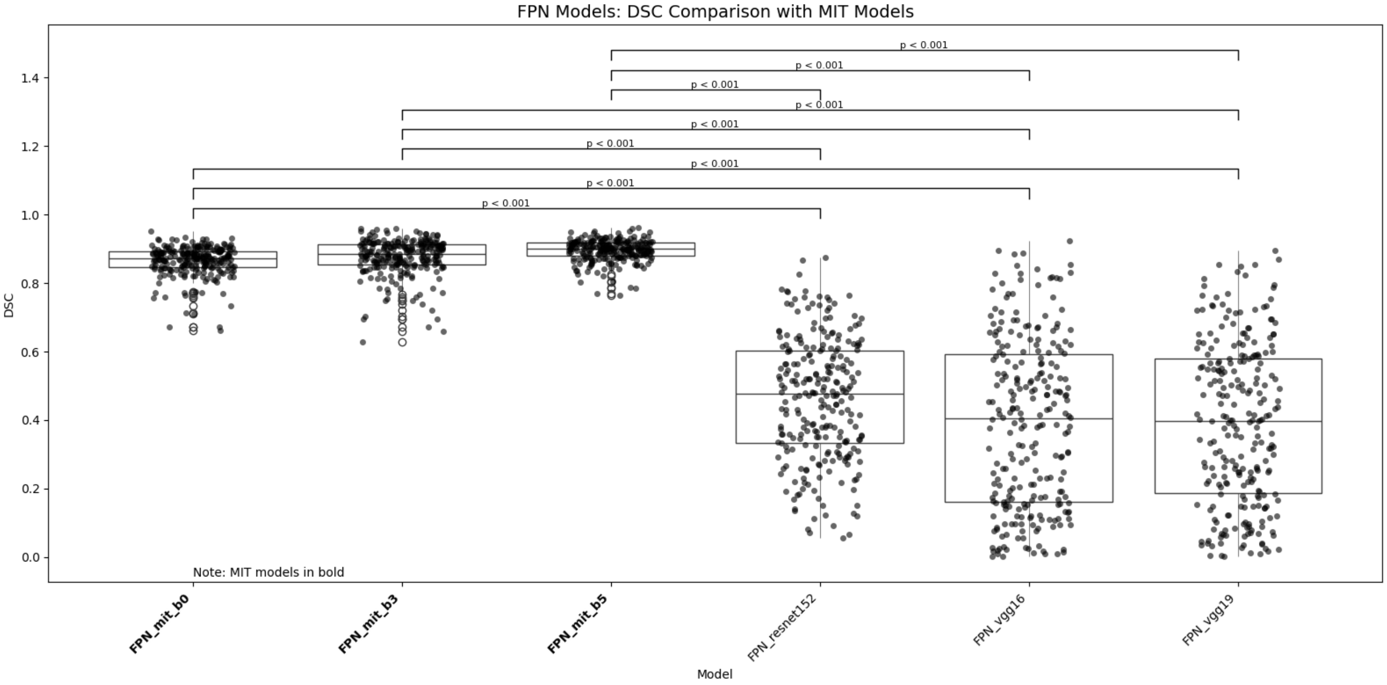

Figure S8. Boxplot of Dice Similarity Coefficient (DSC) distributions for FPN models under high-noise condition for Proton MRI. The dramatic performance decline in CNN-based FPN models under high noise conditions demonstrates the critical advantage of transformer architectures in challenging imaging scenarios typical of clinical practice.

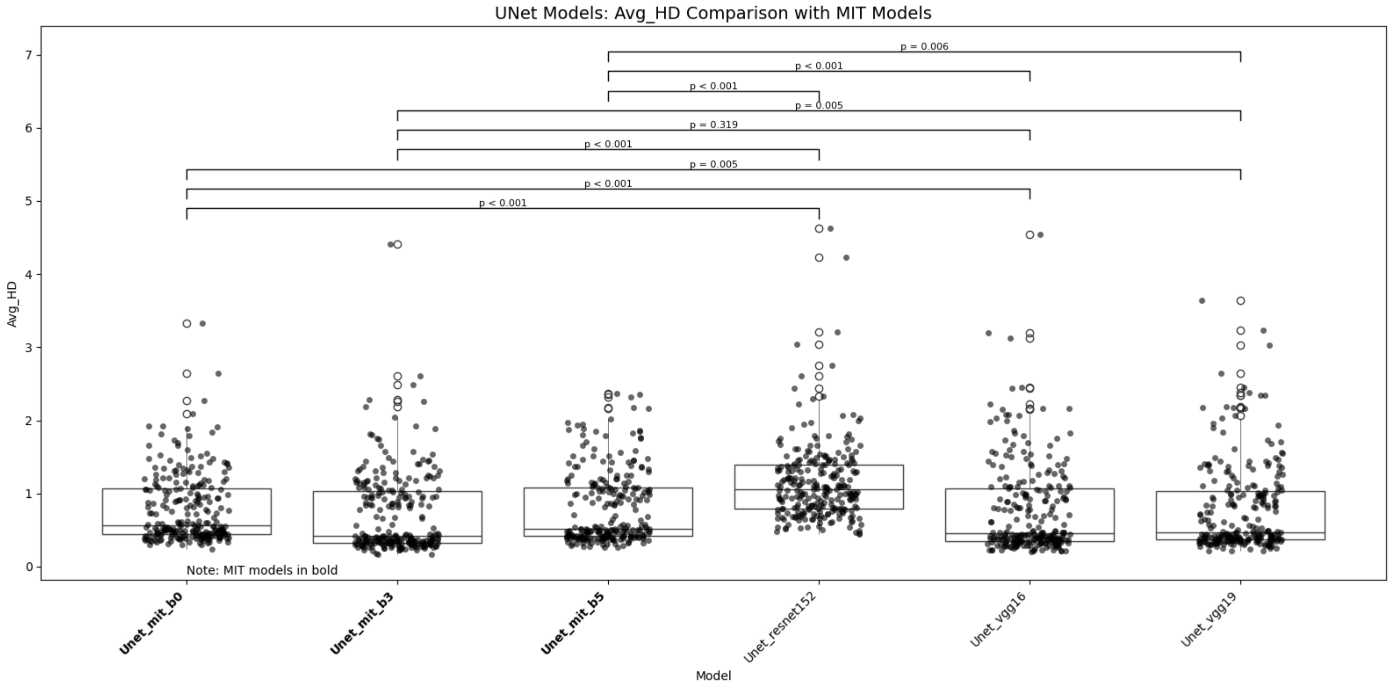

Figure S9. Boxplot of Average Hausdorff Distance (Avg HD) distributions for UNet models under no-noise condition for proton MRI. Boundary accuracy metrics show minimal differences between model types under optimal conditions, with all architectures achieving low Avg HD values indicative of precise segmentation boundaries.

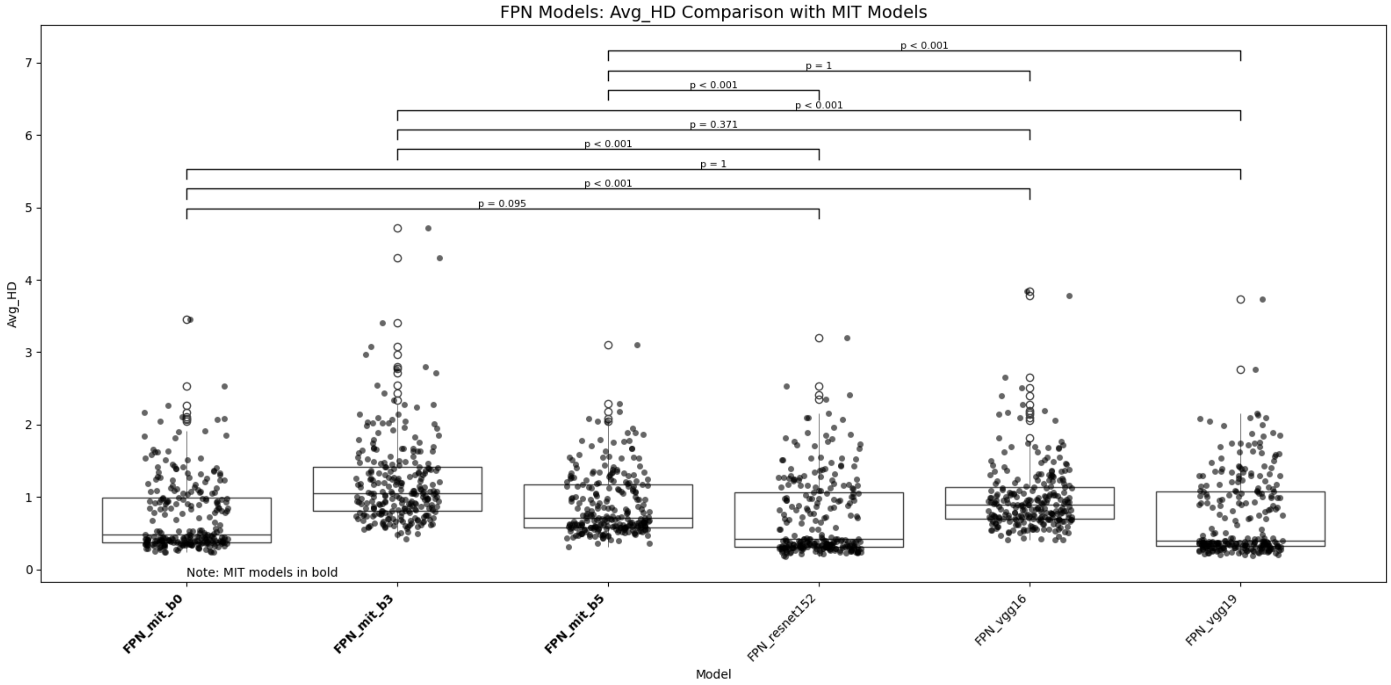

Figure S10. Boxplot of Average Hausdorff Distance (Avg HD) distributions for FPN models under no-noise condition for proton MRI. FPN models demonstrate excellent boundary delineation in noise-free conditions, with slight advantages observed in ViT-based variants that maintain more consistent edge detection performance.

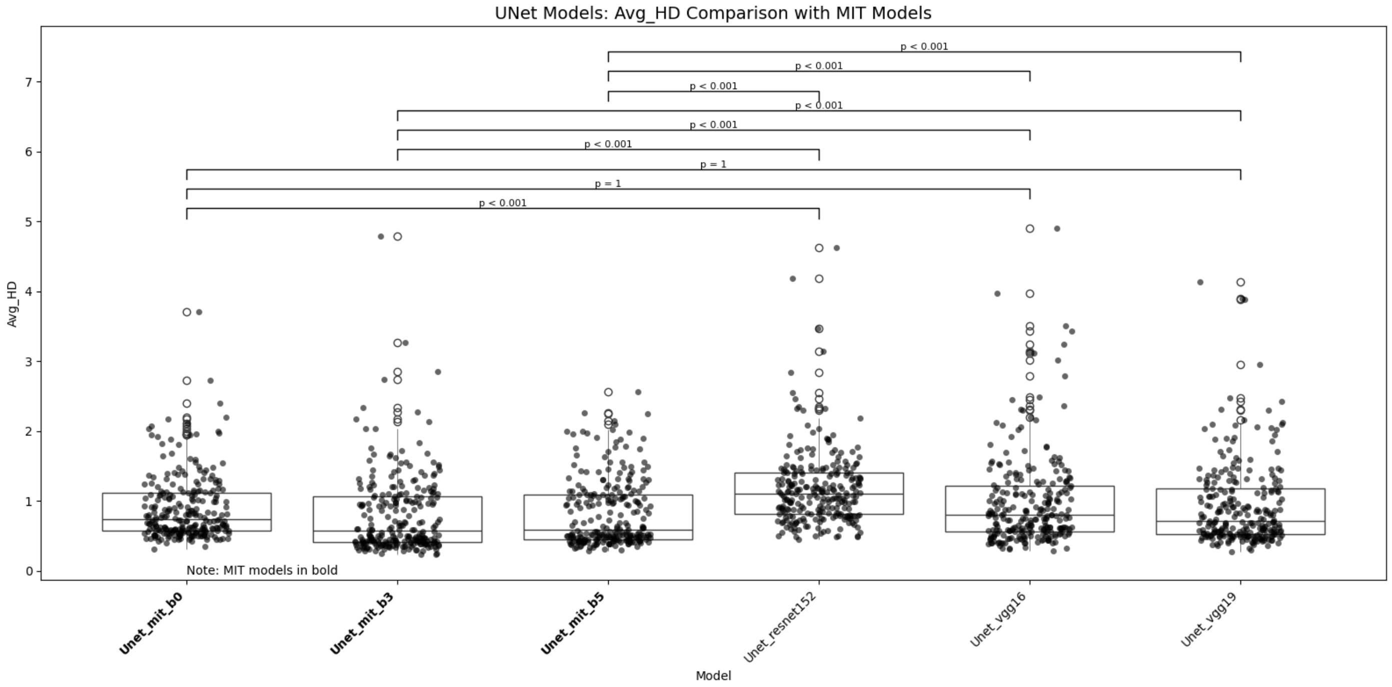

Figure S11. Boxplot of Average Hausdorff Distance (Avg HD) distributions for UNet models under low-noise condition for proton MRI. As noise is introduced, ViT-based UNet models show superior boundary preservation compared to CNN variants, with lower median Avg HD values and reduced outlier presence indicating more reliable edge detection.

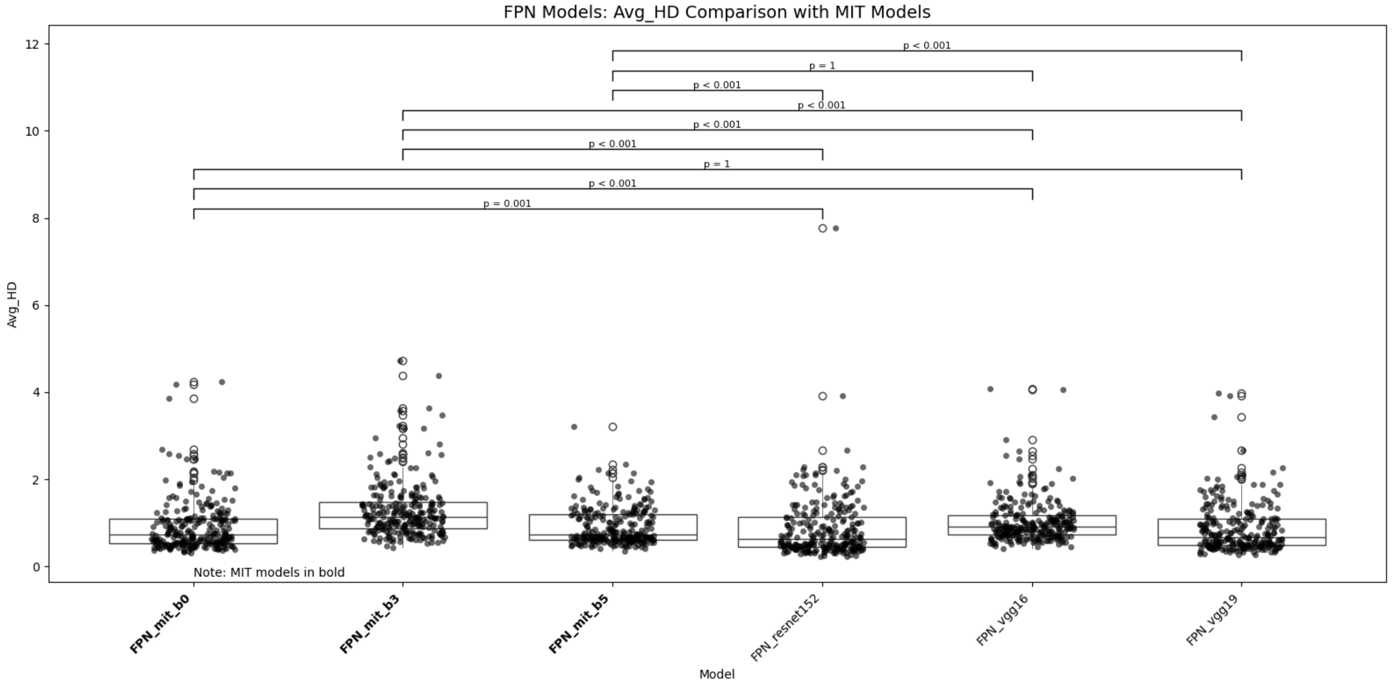

Figure S12. Boxplot of Average Hausdorff Distance (Avg HD) distributions for FPN models under low-noise condition for proton MRI. The enhanced boundary accuracy of transformer-based FPN models under noise conditions demonstrates their ability to maintain spatial precision even when local image features are corrupted.

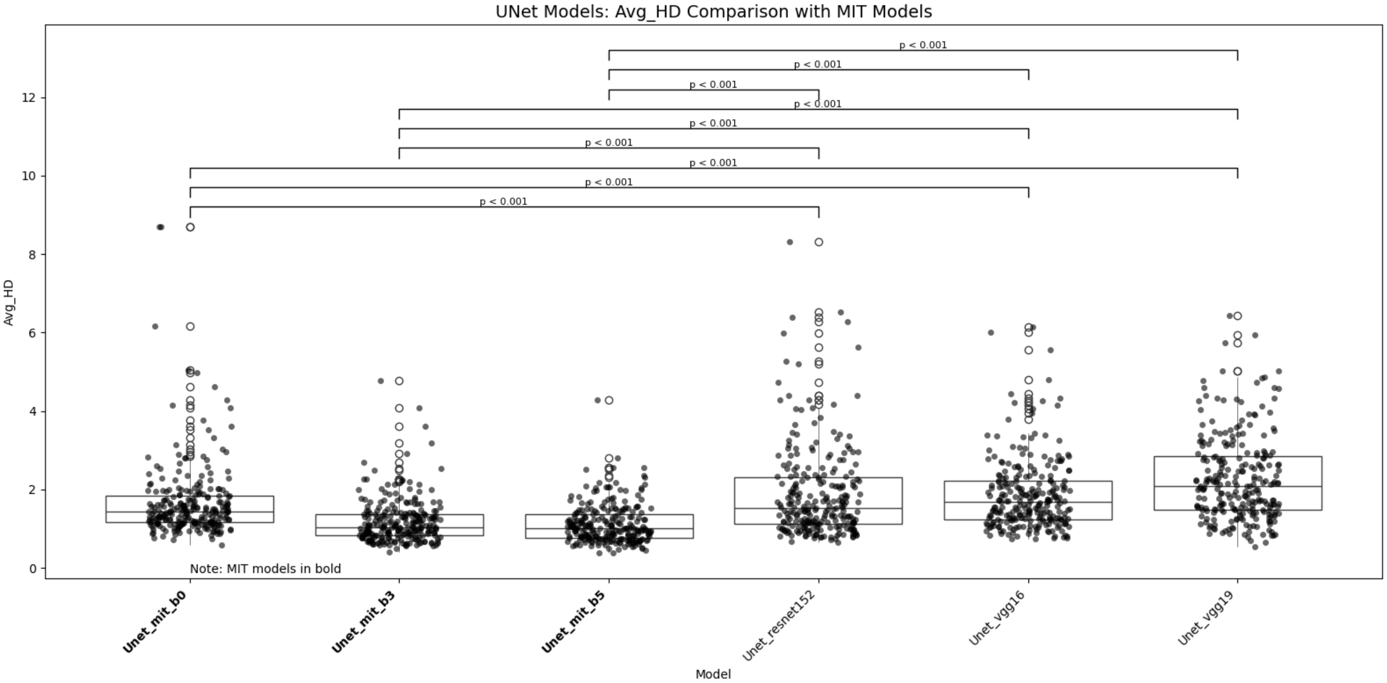

Figure S13. Boxplot of Average Hausdorff Distance (Avg HD) distributions for UNet models under medium-noise condition for proton MRI. Medium noise levels reveal significant differences in boundary accuracy, with CNN-based models showing increased Avg HD values and greater variability, while ViT models maintain tighter error distributions.

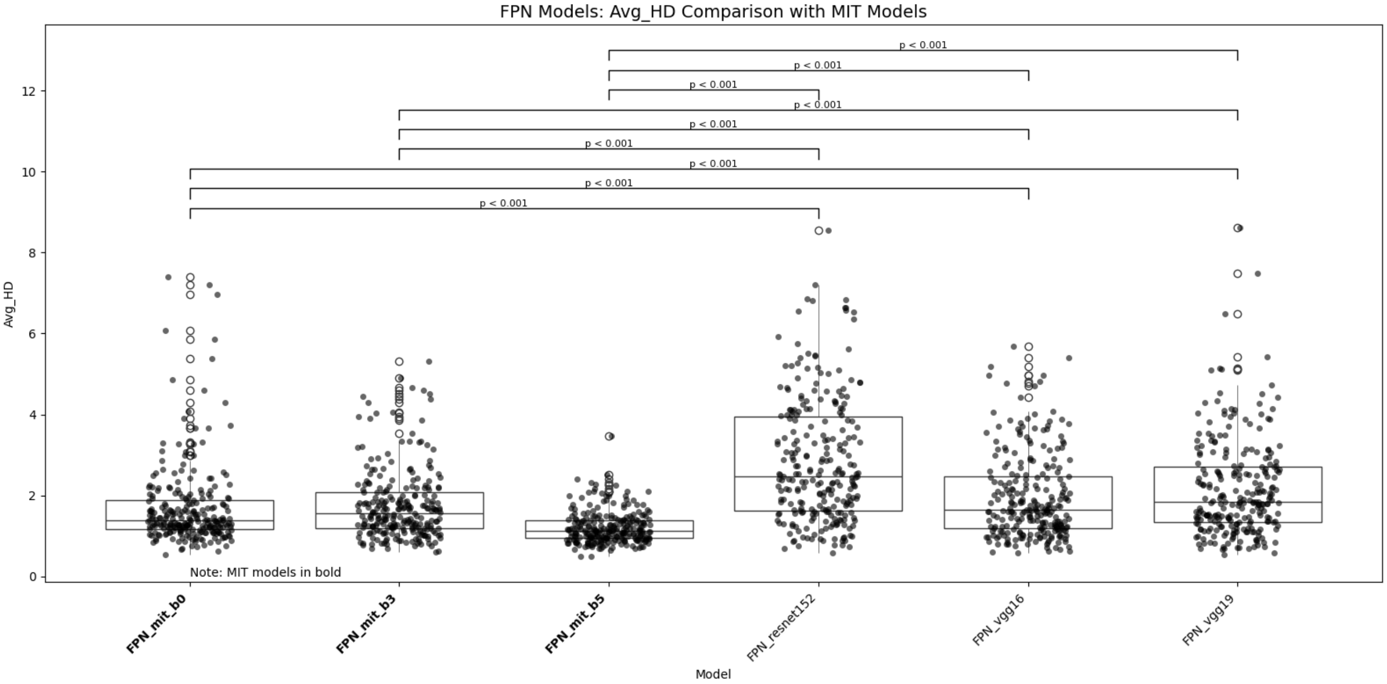

Figure S14. Boxplot of Average Hausdorff Distance (Avg HD) distributions for FPN models under medium-noise condition for proton MRI. The growing performance gap between ViT and CNN architectures in boundary-sensitive metrics underscores the clinical importance of model selection in noisy imaging environments.

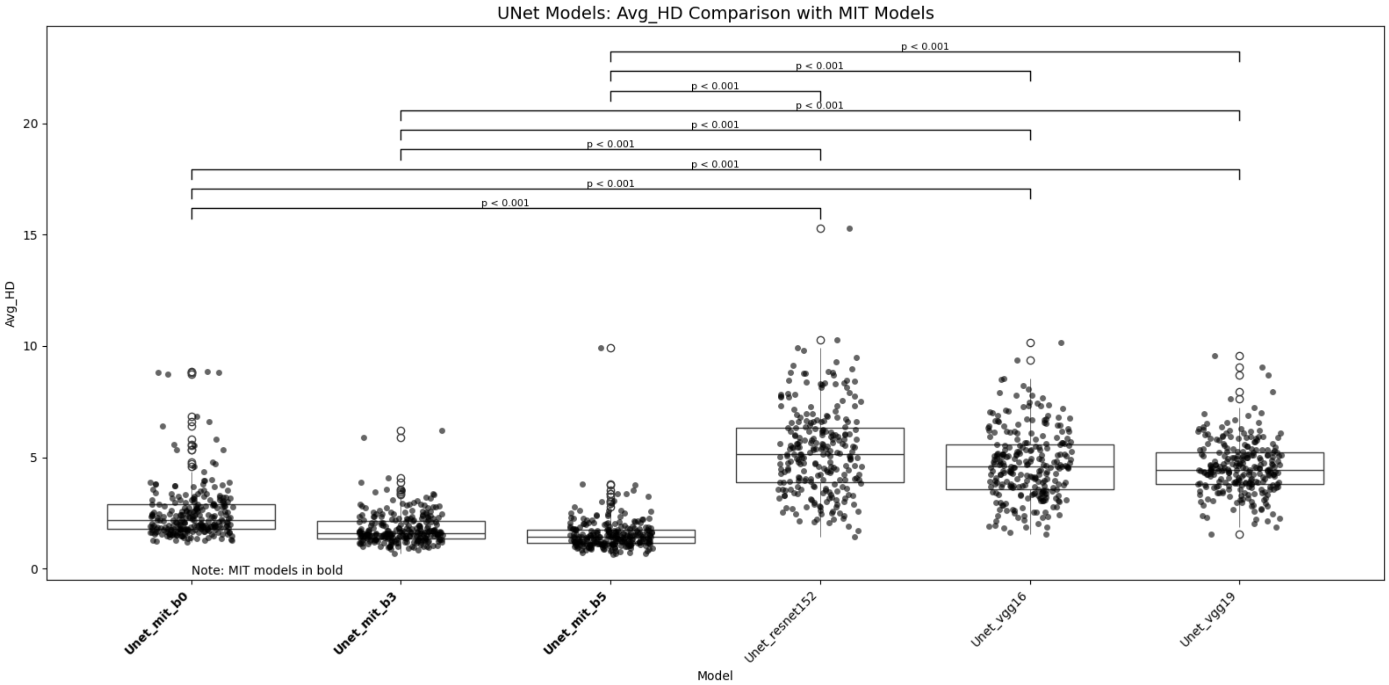

Figure S15. Boxplot of Average Hausdorff Distance (Avg HD) distributions for UNet models under high-noise condition for proton MRI. Under severe noise conditions, CNN models exhibit substantial boundary detection failures with high Avg HD values and extreme outliers, while transformer models demonstrate remarkable resilience in maintaining anatomical boundary integrity.

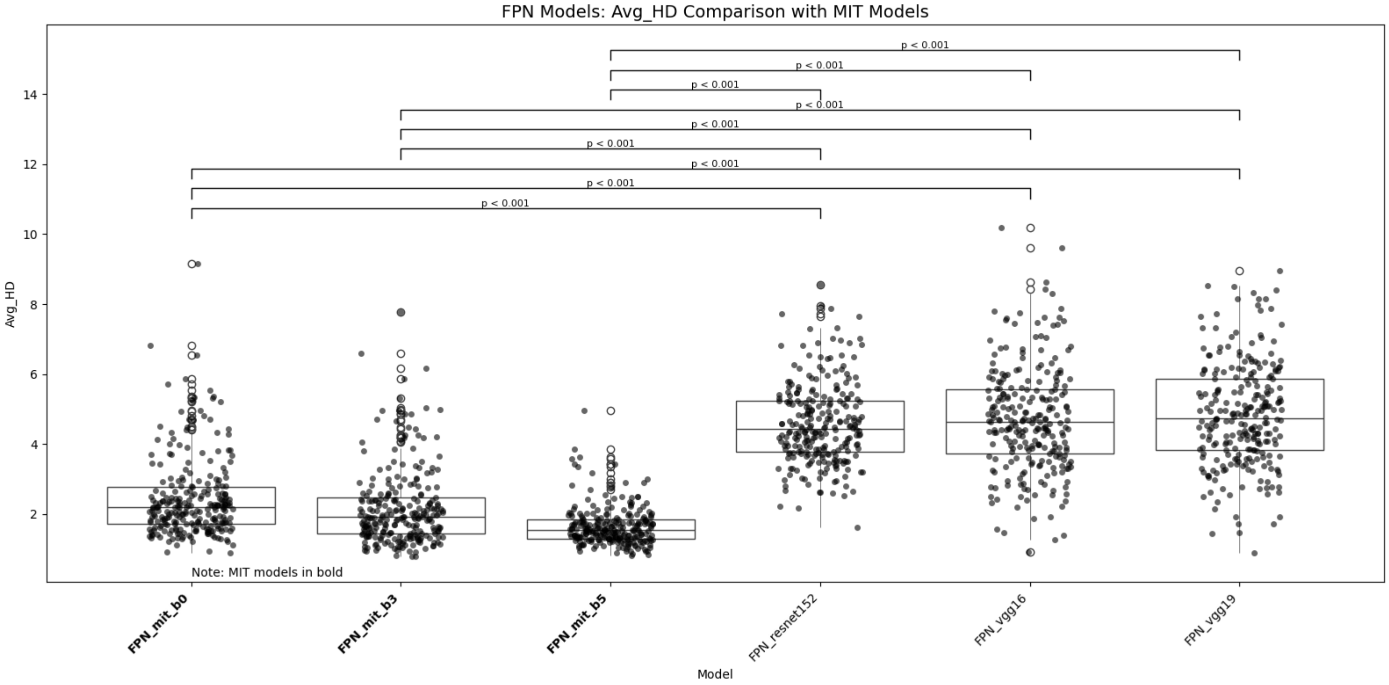

Figure S16. Boxplot of Average Hausdorff Distance (Avg HD) distributions for FPN models under high-noise condition for proton MRI. The superior boundary preservation of ViT-based FPN models under challenging conditions highlights their potential for deployment in clinical scenarios where image quality may be suboptimal.

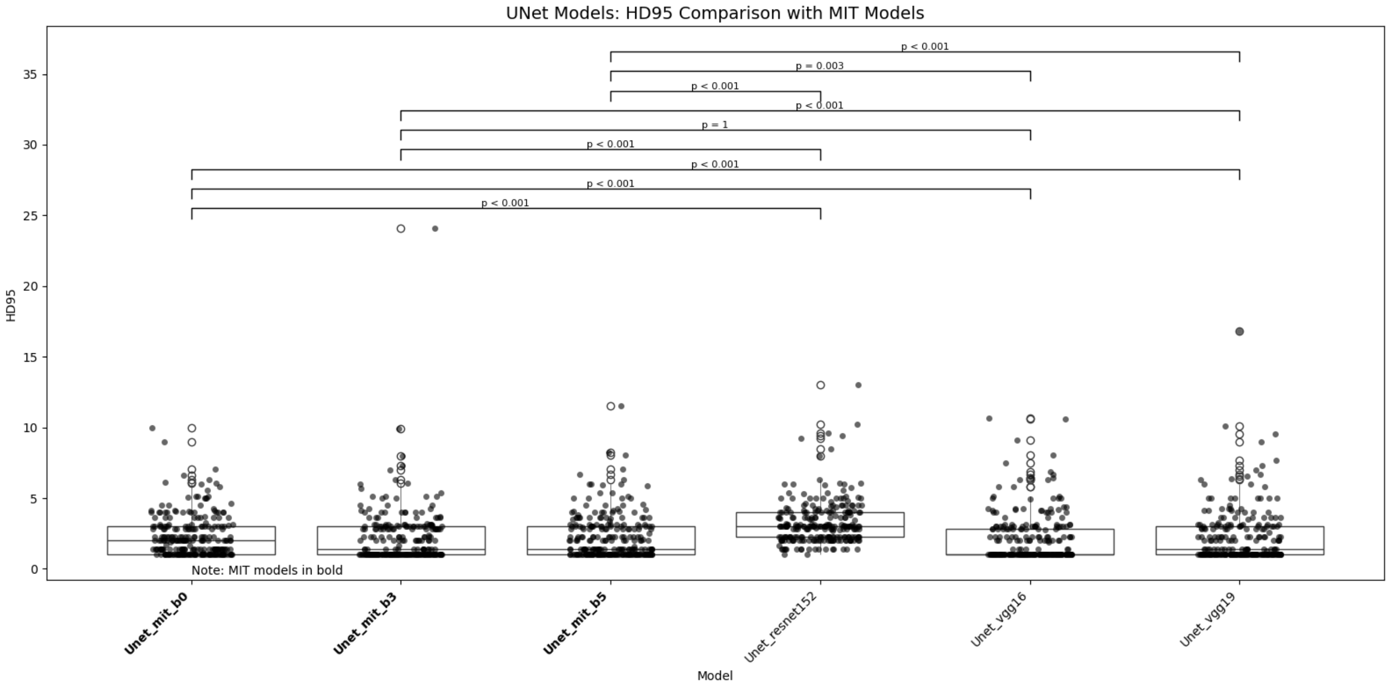

Figure S17. Boxplot of Hausdorff Distance at the 95th percentile (HD95) distributions for UNet models under no-noise condition for proton MRI. HD95 metrics in noise-free conditions show comparable performance across model types, with all architectures achieving low values indicative of accurate boundary delineation without significant outlier errors.

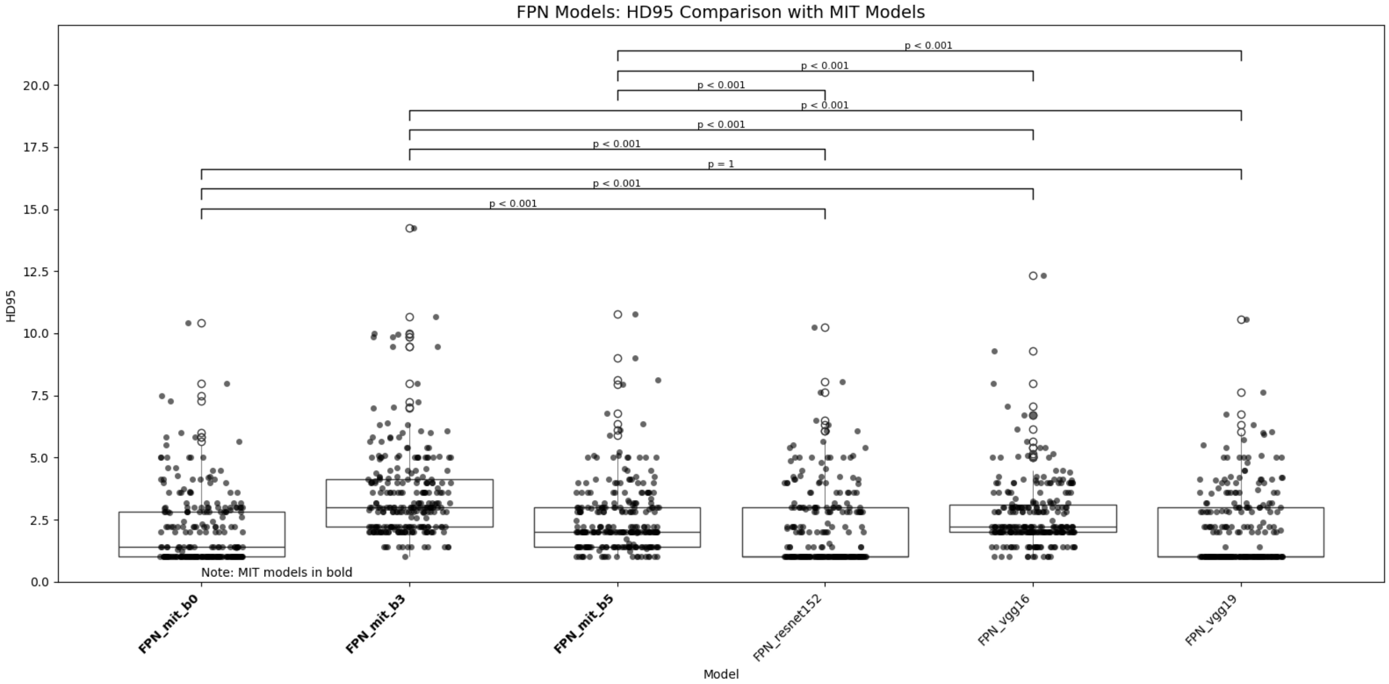

Figure S18. Boxplot of Hausdorff Distance at the 95th percentile (HD95) distributions for FPN models under no-noise condition for proton MRI. Under optimal imaging conditions, FPN models demonstrate excellent performance in controlling worst-case boundary errors, with minimal differences between CNN and ViT-based approaches.

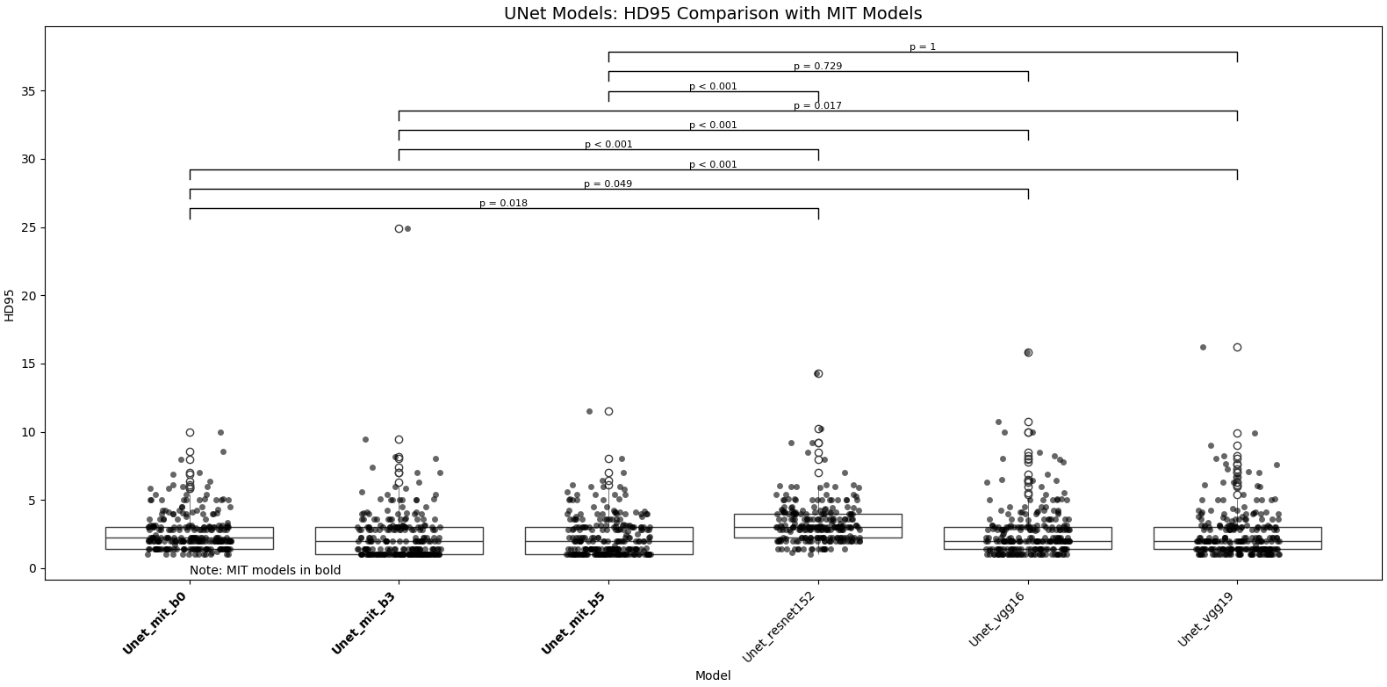

Figure S19. Boxplot of Hausdorff Distance at the 95th percentile (HD95) distributions for UNet models under low-noise condition for proton MRI. Introduction of noise begins to differentiate model performance in controlling maximum boundary errors, with ViT-based models showing improved outlier control and more consistent HD95 values.

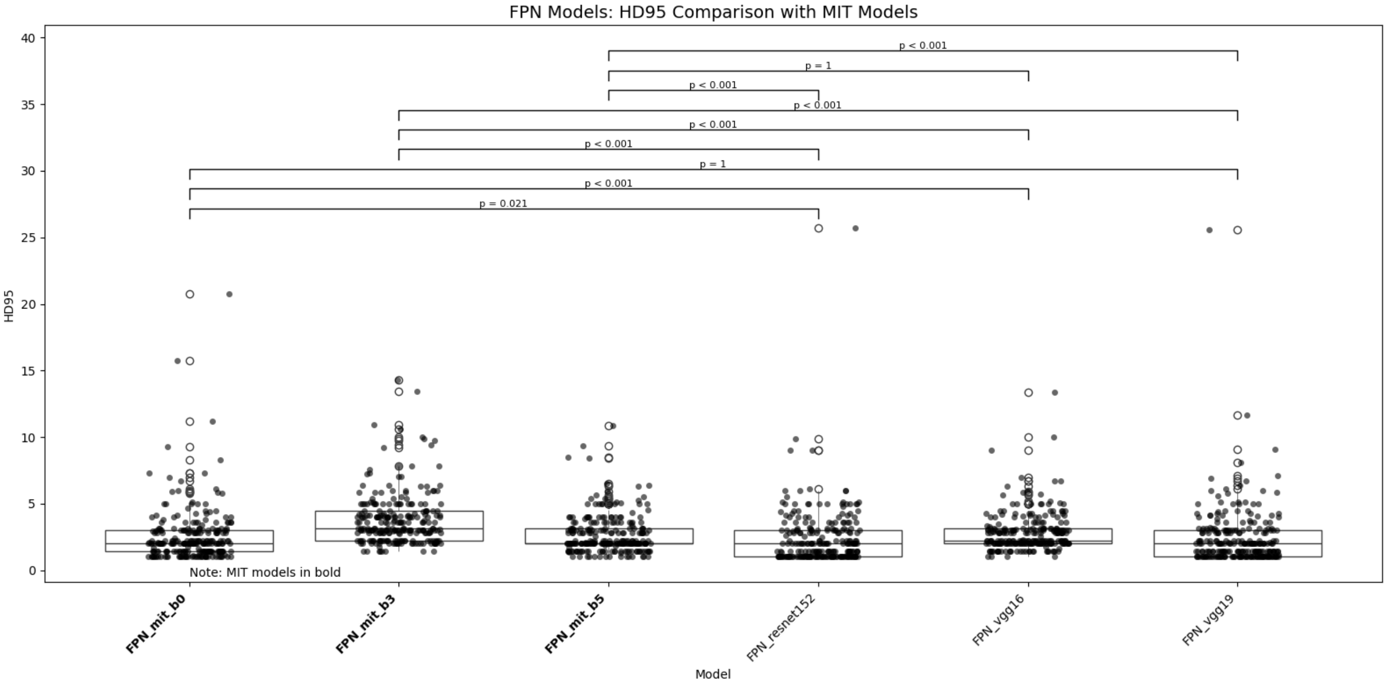

Figure S20. Boxplot of Hausdorff Distance at the 95th percentile (HD95) distributions for FPN models under low-noise condition for proton MRI. Transformer-based FPN models demonstrate superior ability to control worst-case segmentation errors under noise, indicating more reliable clinical performance.

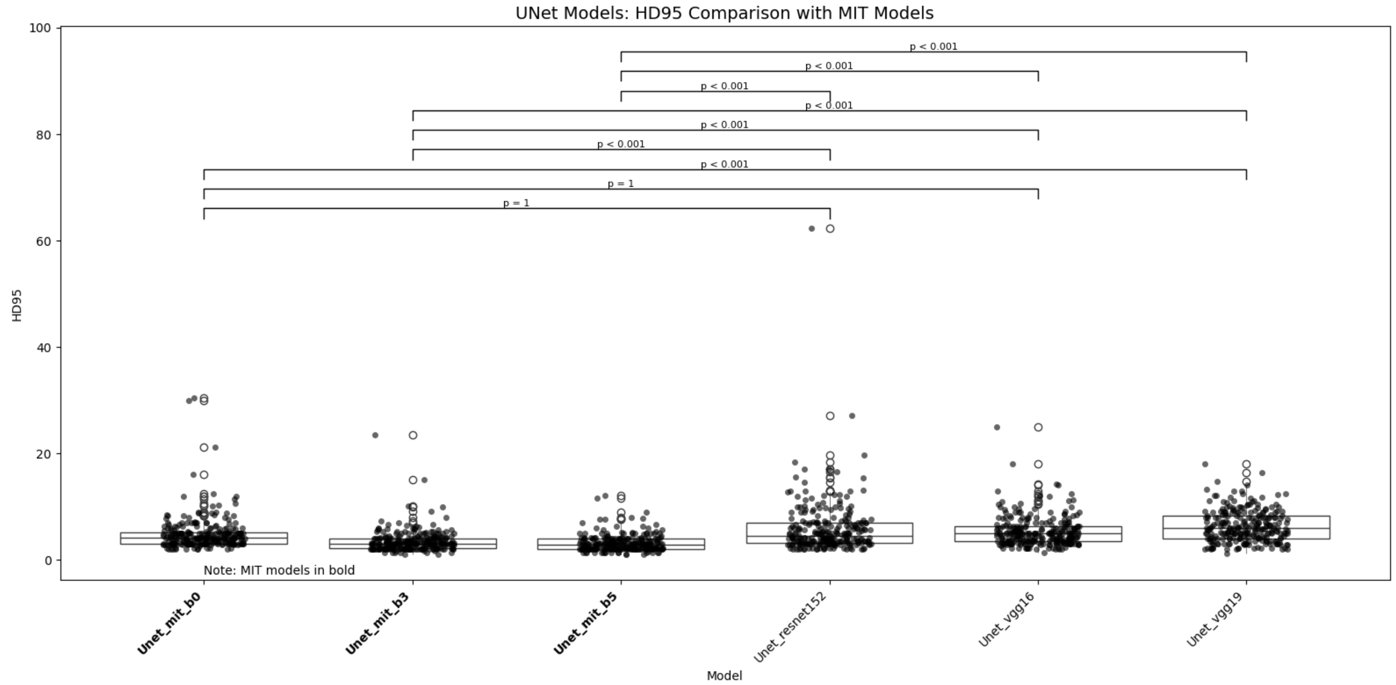

Figure S21. Boxplot of Hausdorff Distance at the 95th percentile (HD95) distributions for UNet models under medium-noise condition for proton MRI. Medium noise levels reveal significant advantages of ViT architectures in managing extreme boundary errors, with CNN models showing increased HD95 values and wider error distributions.

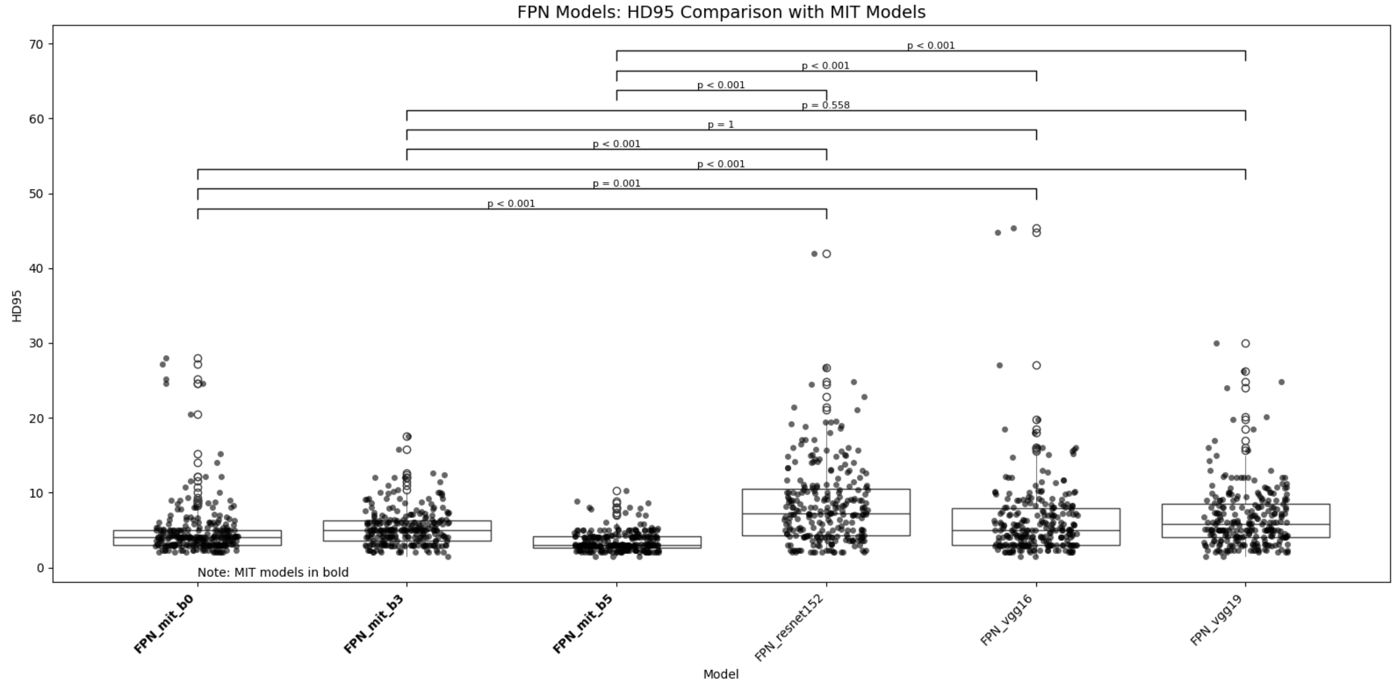

Figure S22. Boxplot of Hausdorff Distance at the 95th percentile (HD95) distributions for FPN models under medium-noise condition for proton MRI. The enhanced outlier control of transformer-based models becomes increasingly important as noise levels rise, directly impacting clinical measurement reliability.

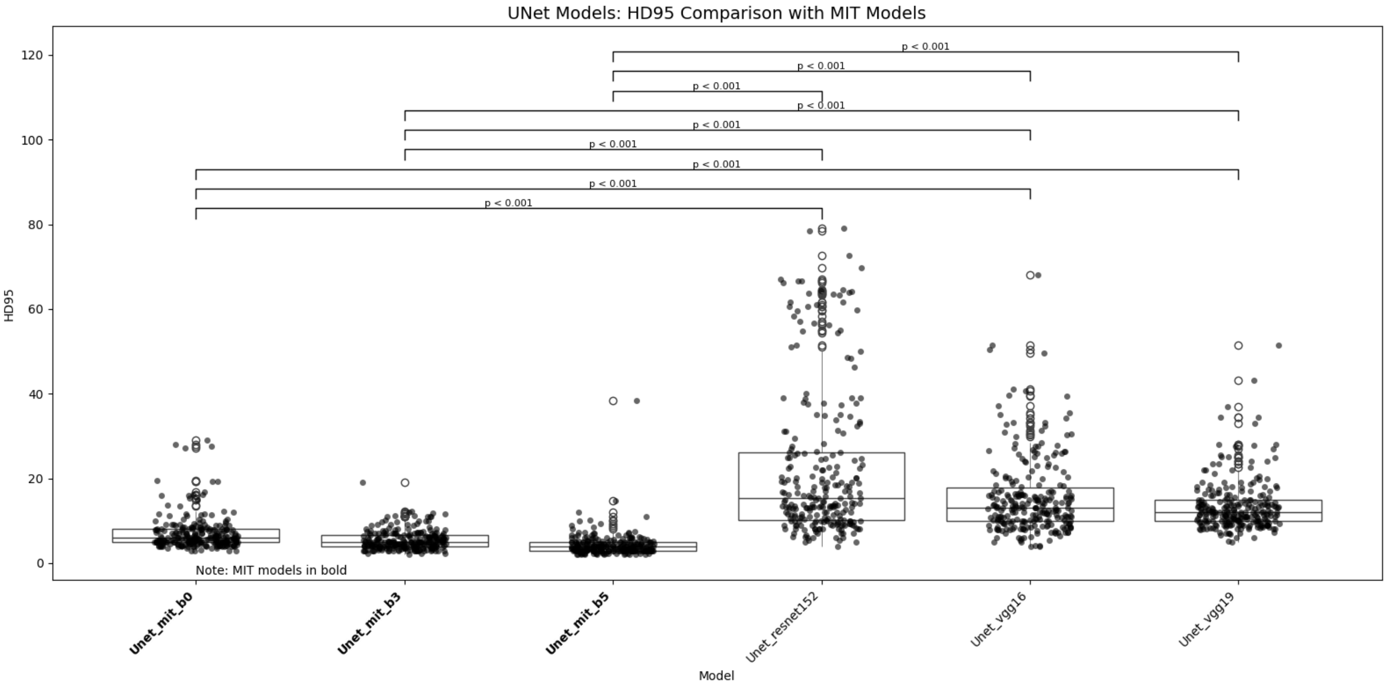

Figure S23. Boxplot of Hausdorff Distance at the 95th percentile (HD95) distributions for UNet models under high-noise condition for proton MRI. Under severe noise conditions, CNN models suffer from extreme HD95 outliers indicating catastrophic boundary failures, while ViT models maintain controlled error distributions essential for clinical deployment.

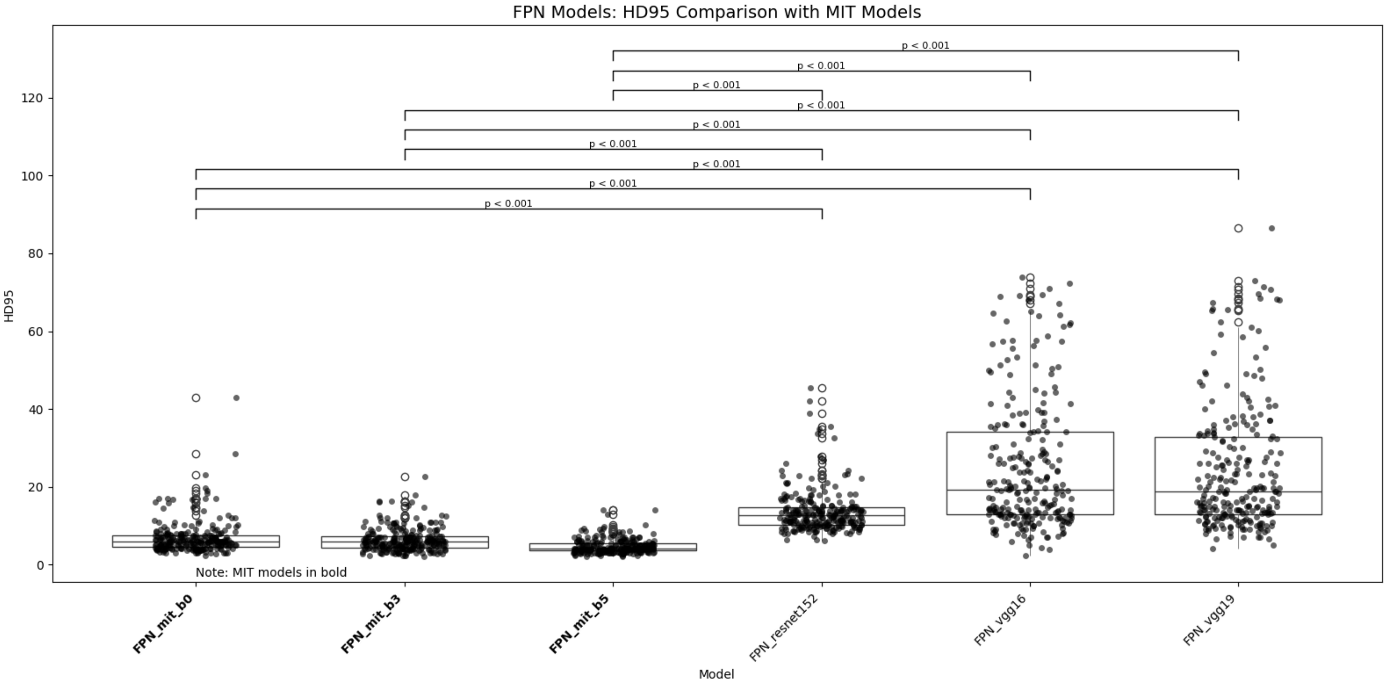

Figure S24. Boxplot of Hausdorff Distance at the 95th percentile (HD95) distributions for FPN models under high-noise condition for proton MRI. The dramatic difference in outlier control between ViT and CNN architectures under challenging conditions demonstrates the critical importance of model architecture selection for robust clinical applications.

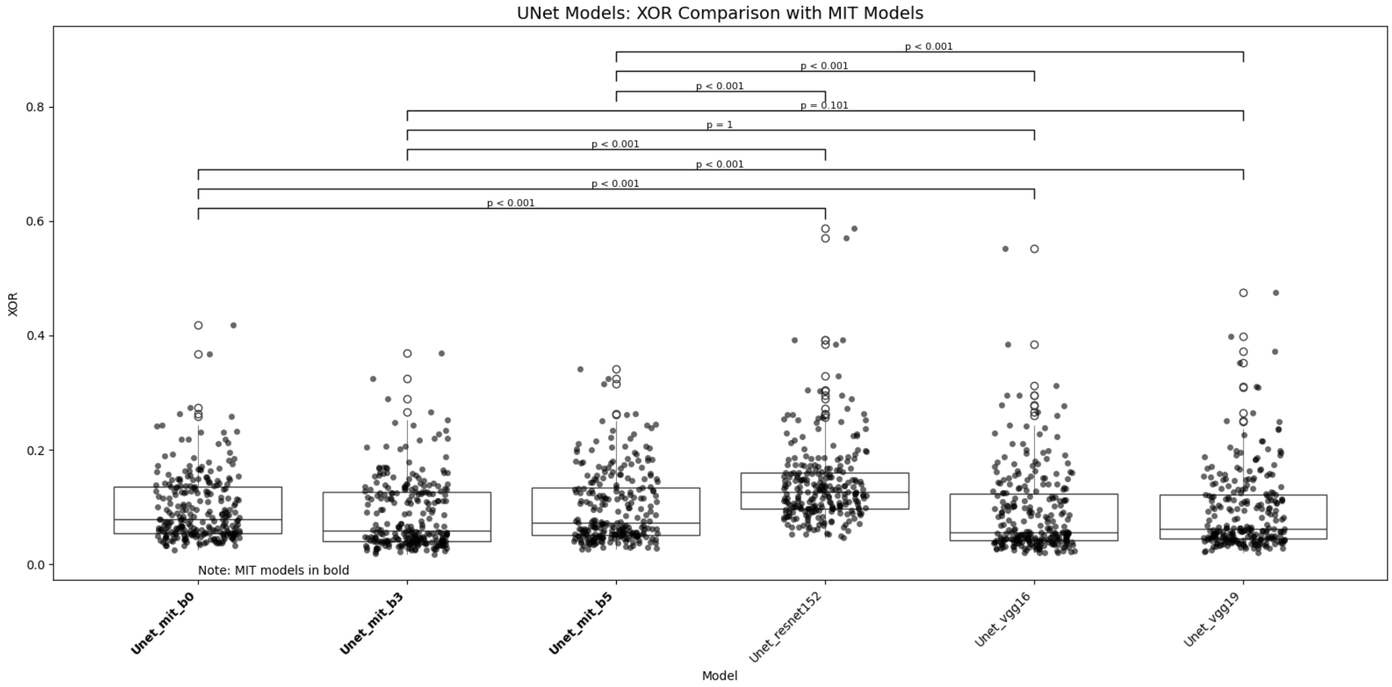

Figure S25. Boxplot of XOR Error distributions for UNet models under no-noise condition for proton MRI. Pixel-level accuracy metrics show excellent performance across all model types in noise-free conditions, with minimal XOR errors indicating high fidelity segmentation masks.

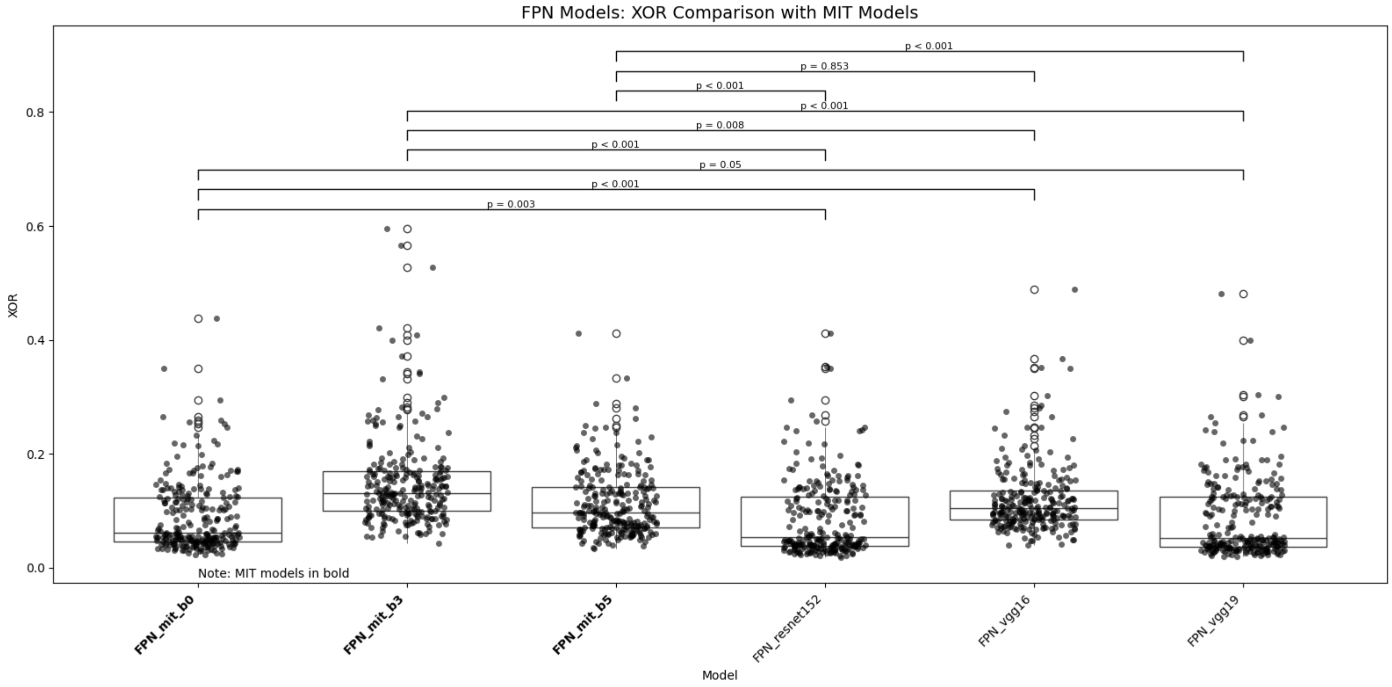

Figure S26. Boxplot of XOR Error distributions for FPN models under no-noise condition for proton MRI. Under optimal imaging conditions, FPN architectures achieve low XOR error rates with slight advantages observed in ViT-based variants that demonstrate more consistent pixel-level accuracy.

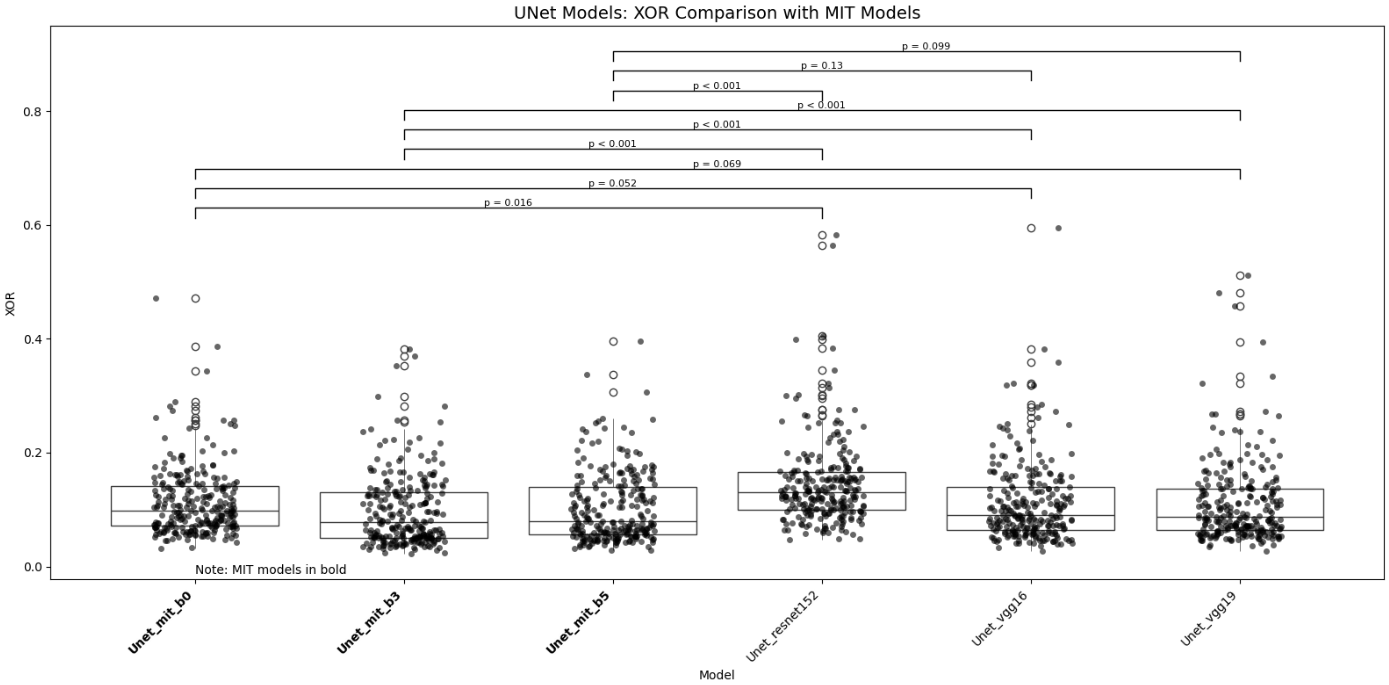

Figure S27. Boxplot of XOR Error distributions for UNet models under low-noise condition for proton MRI. As noise is introduced, ViT-based models maintain superior pixel-level accuracy with lower XOR error rates and tighter distributions compared to CNN counterparts.

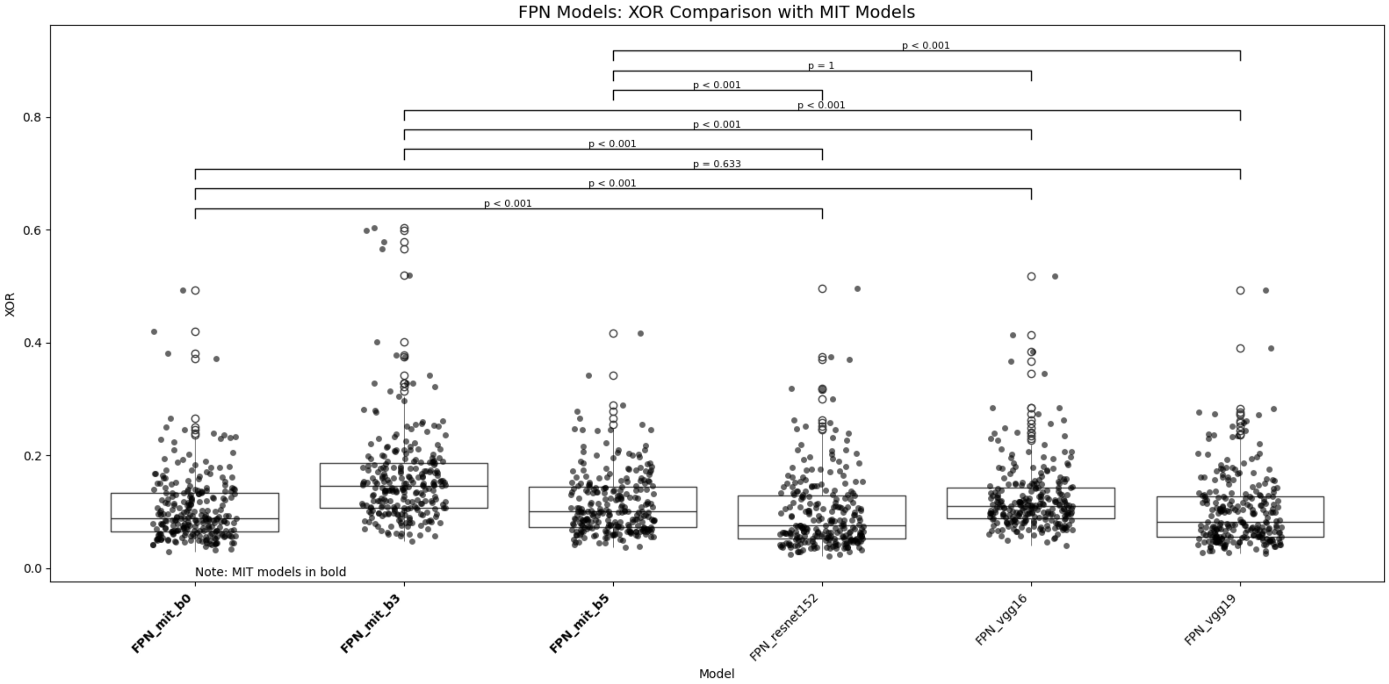

Figure S28. Boxplot of XOR Error distributions for FPN models under low-noise condition for proton MRI. The enhanced pixel-level precision of transformer-based FPN models under noise conditions reflects their superior ability to distinguish true anatomical structures from noise artifacts.

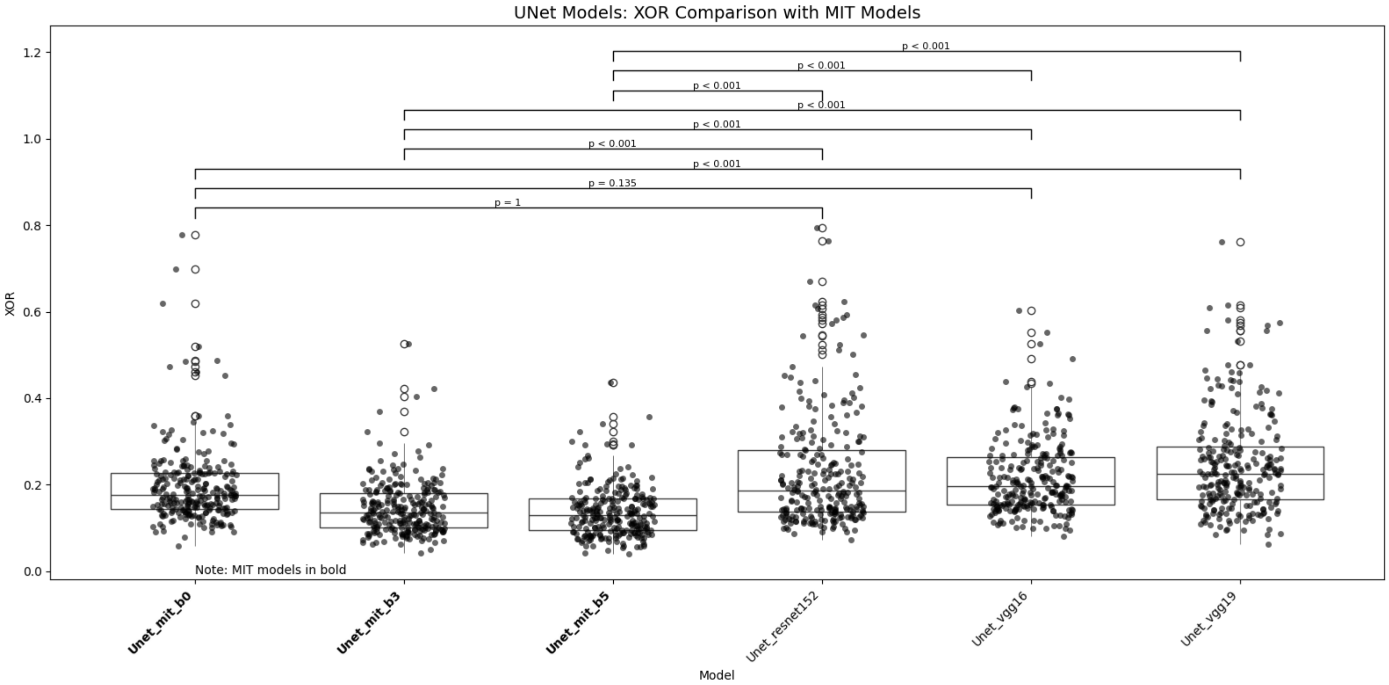

Figure S29. Boxplot of XOR Error distributions for UNet models under medium-noise condition for proton MRI. Medium noise levels significantly impact CNN model accuracy, with increased XOR errors and wider distributions, while ViT models demonstrate remarkable stability in pixel-level classification.

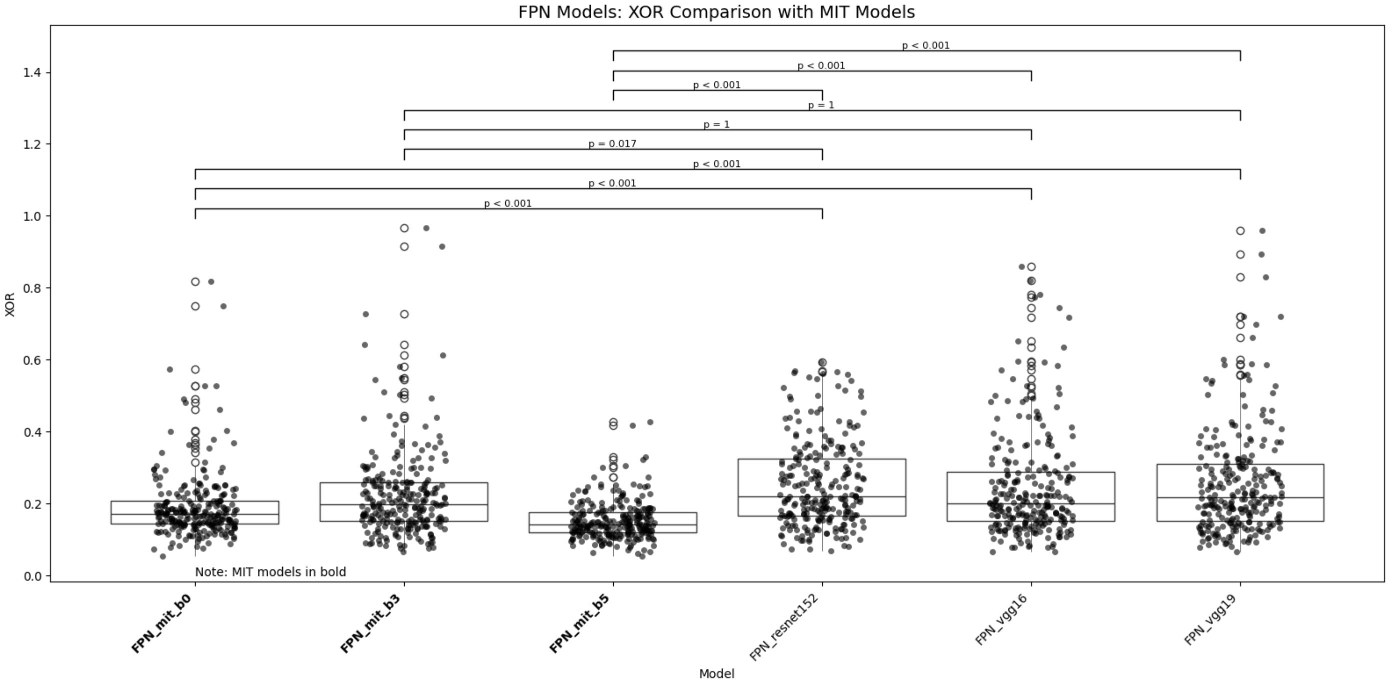

Figure S30. Boxplot of XOR Error distributions for FPN models under medium-noise condition for proton MRI. The growing disparity in XOR error rates between model types underscores the practical advantages of transformer architectures in maintaining segmentation fidelity under realistic clinical conditions.

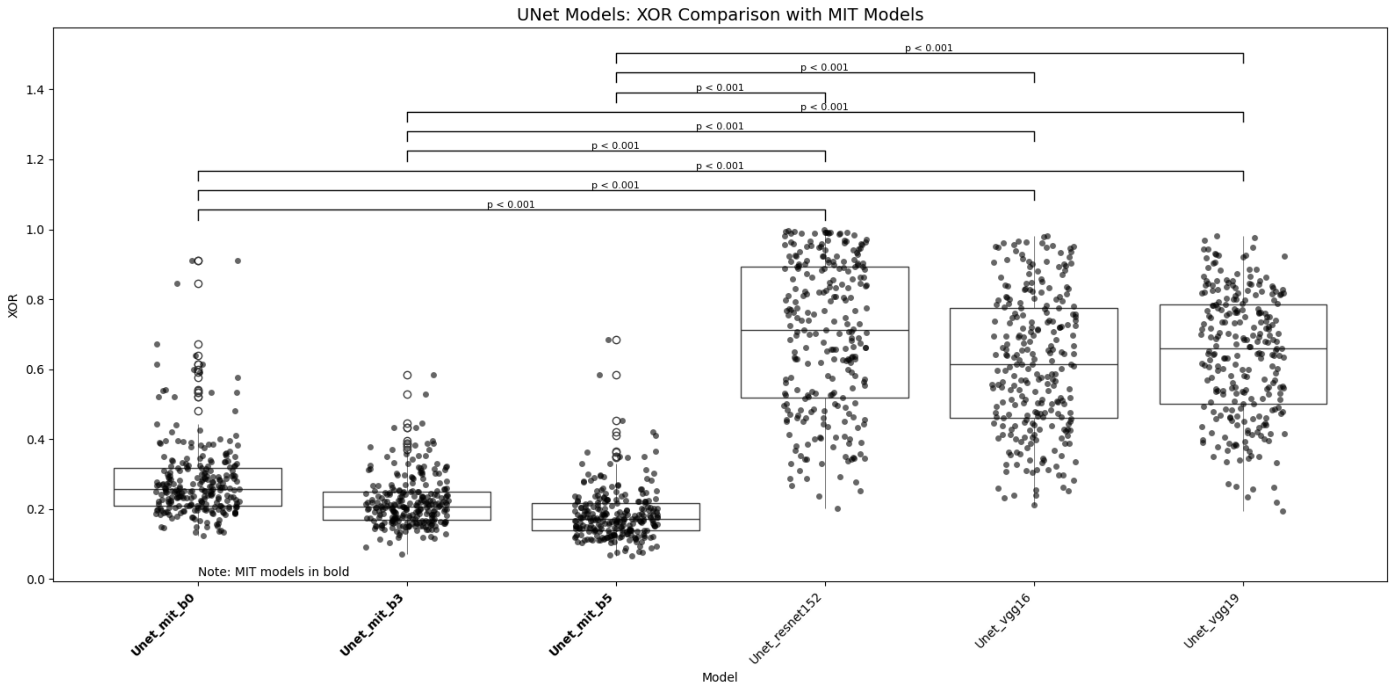

Figure S31. Boxplot of XOR Error distributions for UNet models under high-noise condition for proton MRI. Under challenging noise conditions, CNN models exhibit severe degradation in pixel-level accuracy with high XOR error rates, while ViT models maintain acceptable performance levels critical for clinical utility.

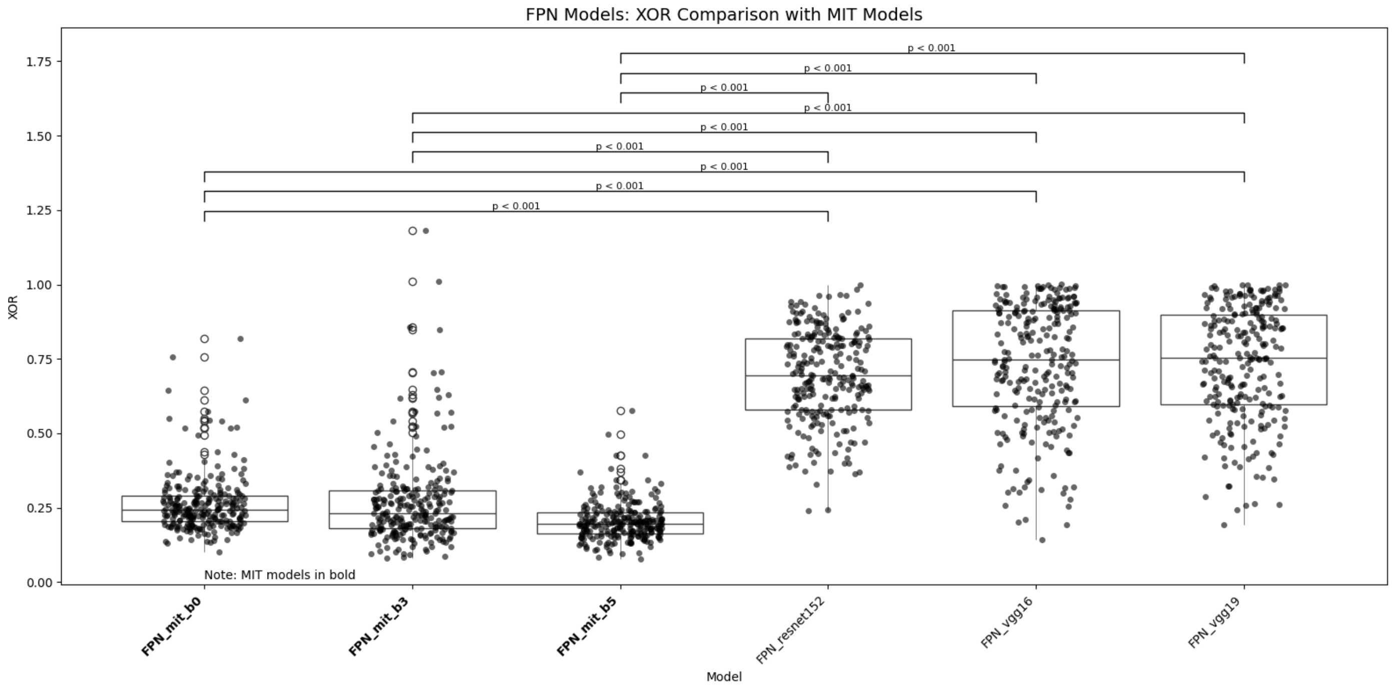

Figure S32. Boxplot of XOR Error distributions for FPN models under high-noise condition for proton MRI. The substantial XOR error differences between ViT and CNN architectures under high noise conditions demonstrate the superior robustness of transformer-based approaches for deployment in challenging imaging environments.

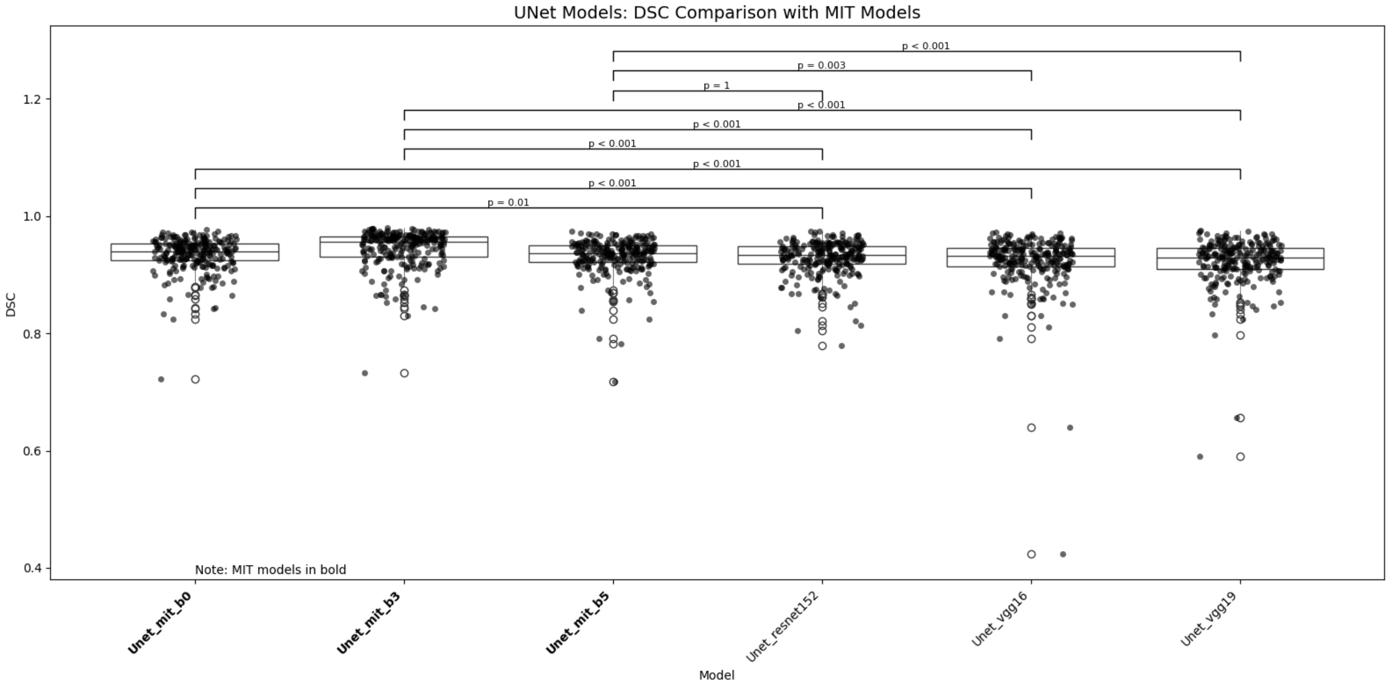

Figure S33. Boxplot of Dice Similarity Coefficient (DSC) distributions for UNet models under no-noise condition for hyperpolarized gas MRI. In the specialized context of hyperpolarized gas MRI, all models achieve strong baseline performance under optimal conditions, with ViT-based architectures showing marginally superior consistency across the diverse patient cohort.

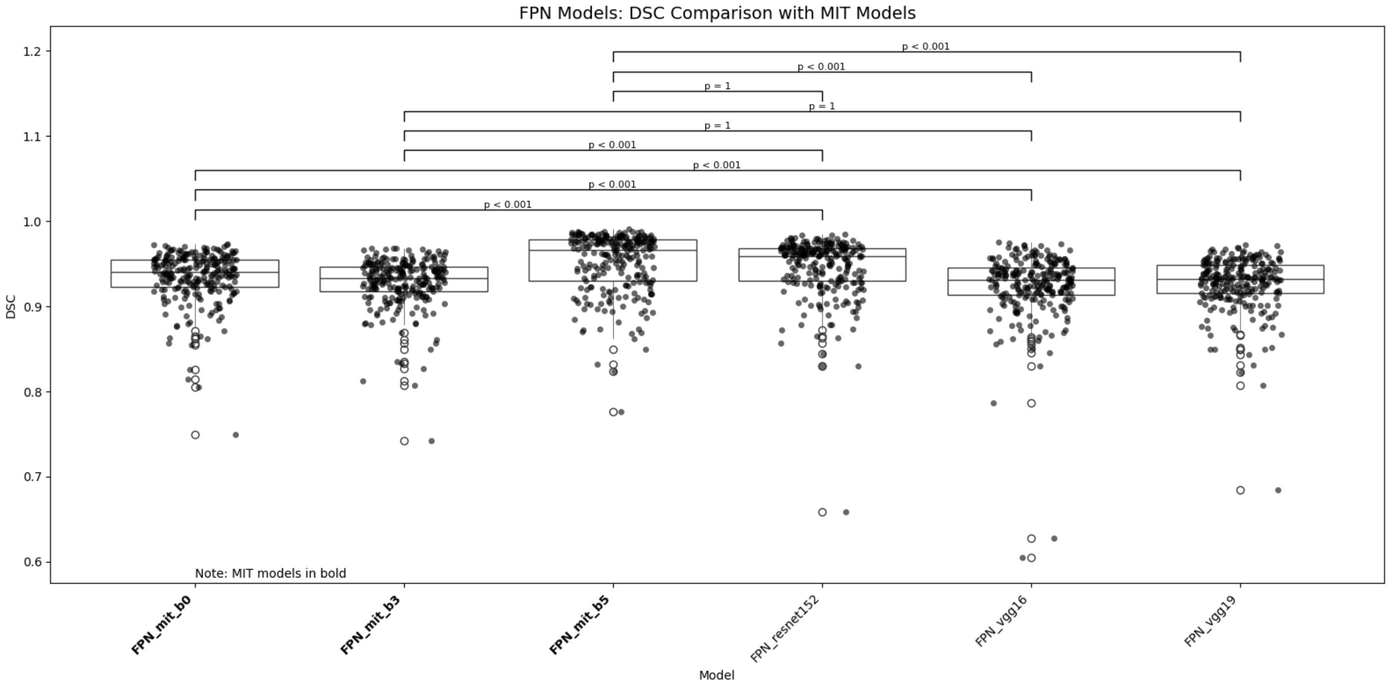

Figure S34. Boxplot of Dice Similarity Coefficient (DSC) distributions for FPN models under no-noise condition for hyperpolarized gas MRI. FPN architectures demonstrate excellent segmentation quality in hyperpolarized gas imaging, with transformer-based models showing slightly tighter performance distributions that may be advantageous for quantitative analysis applications.

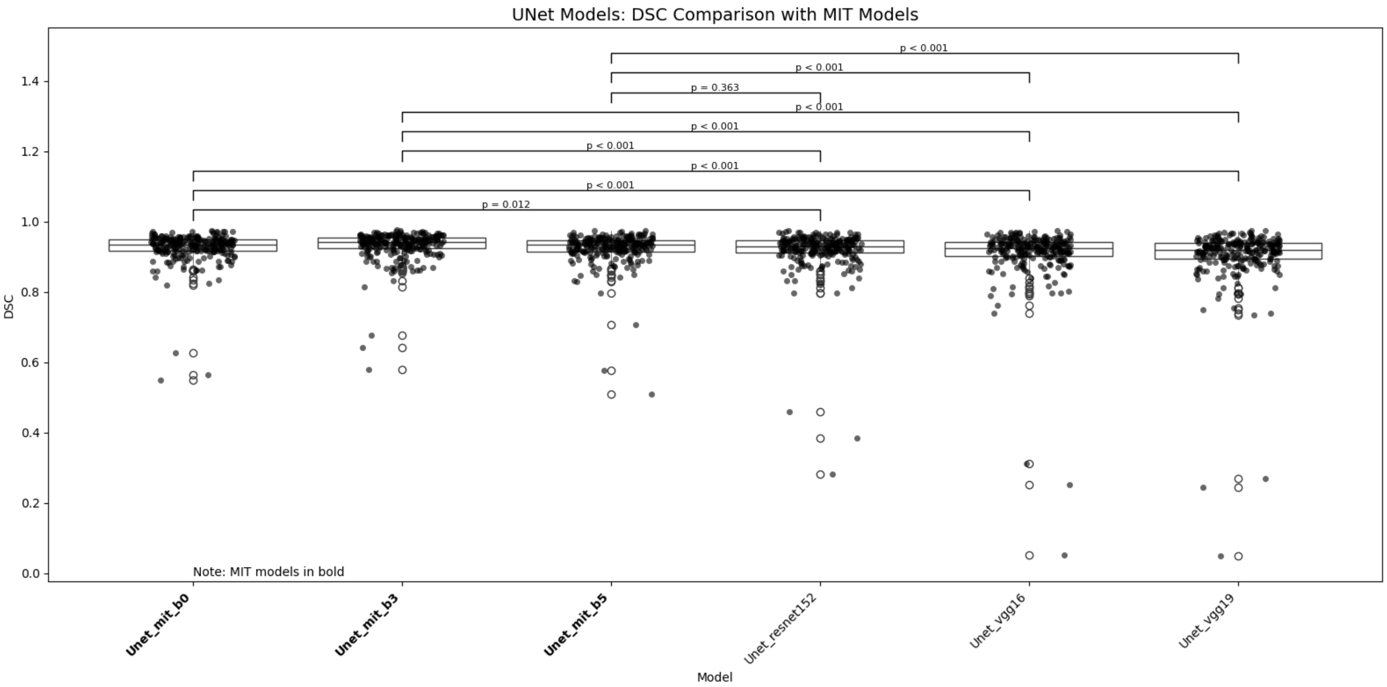

Figure S35. Boxplot of Dice Similarity Coefficient (DSC) distributions for UNet models under low-noise condition for hyperpolarized gas MRI. Given the inherently challenging nature of hyperpolarized gas MRI acquisition, the superior noise tolerance of ViT-based models becomes clinically relevant even at low noise levels, where repeat imaging is typically not feasible.

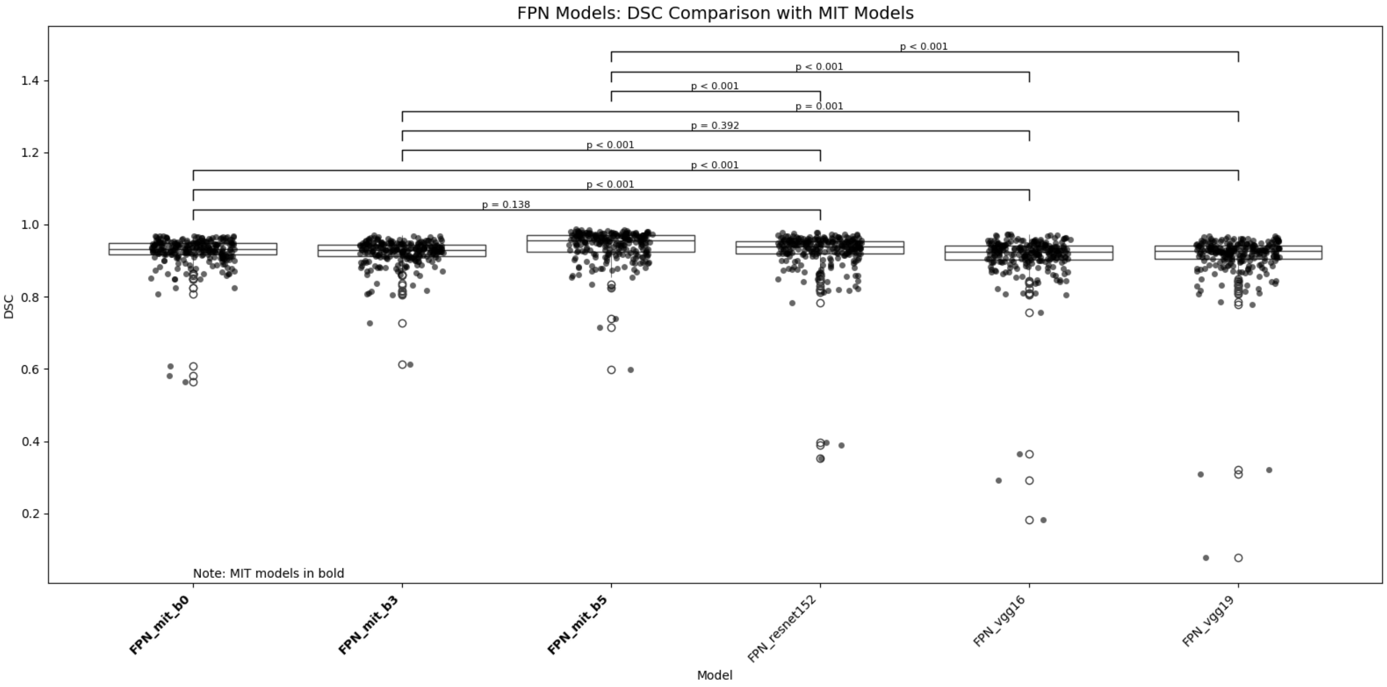

Figure S36. Boxplot of Dice Similarity Coefficient (DSC) distributions for FPN models under low-noise condition for hyperpolarized gas MRI. The enhanced robustness of transformer-based FPN models is particularly valuable in hyperpolarized gas MRI workflows, where breath-hold constraints limit opportunities for image reacquisition.

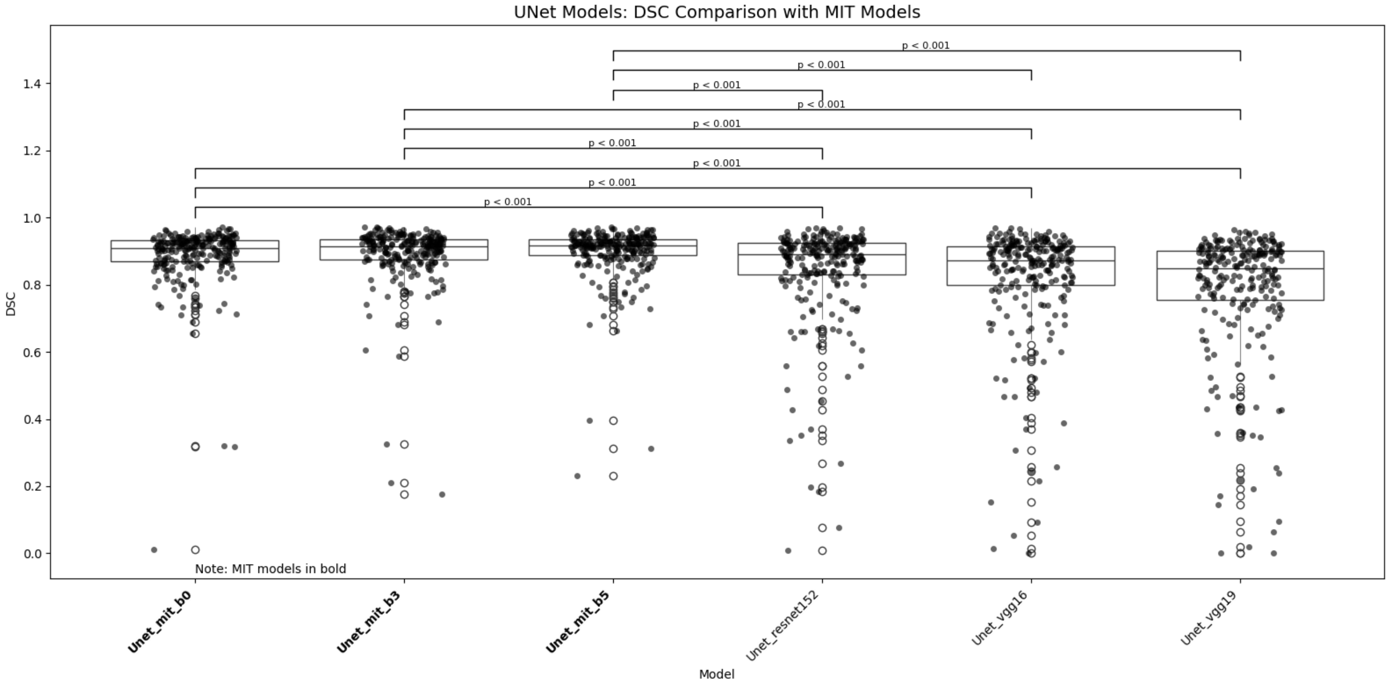

Figure S37. Boxplot of Dice Similarity Coefficient (DSC) distributions for UNet models under medium-noise condition for hyperpolarized gas MRI. Medium noise conditions reveal the clinical advantages of ViT architectures in hyperpolarized gas imaging, where consistent segmentation performance is essential for accurate ventilation defect quantification.

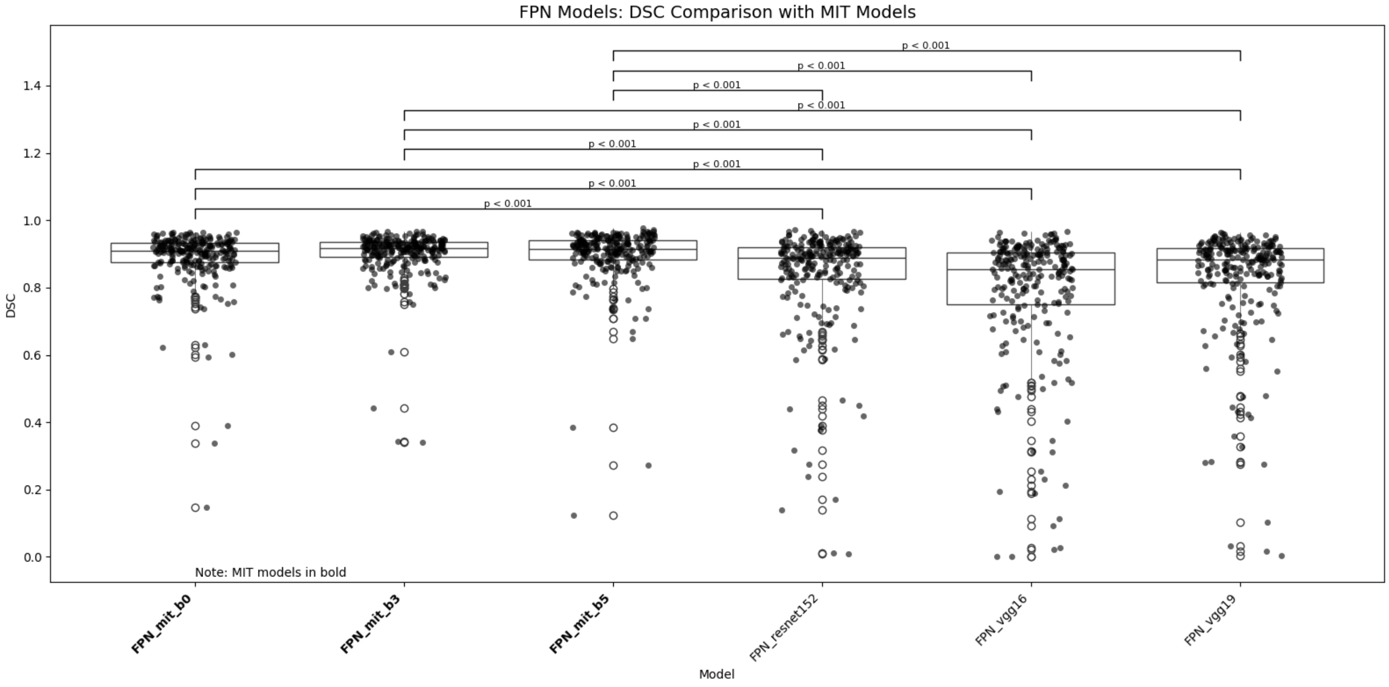

Figure S38. Boxplot of Dice Similarity Coefficient (DSC) distributions for FPN models under medium-noise condition for hyperpolarized gas MRI. The maintained performance of transformer-based models under degraded conditions is crucial for hyperpolarized gas MRI applications, where image quality variations are common due to patient-specific factors.

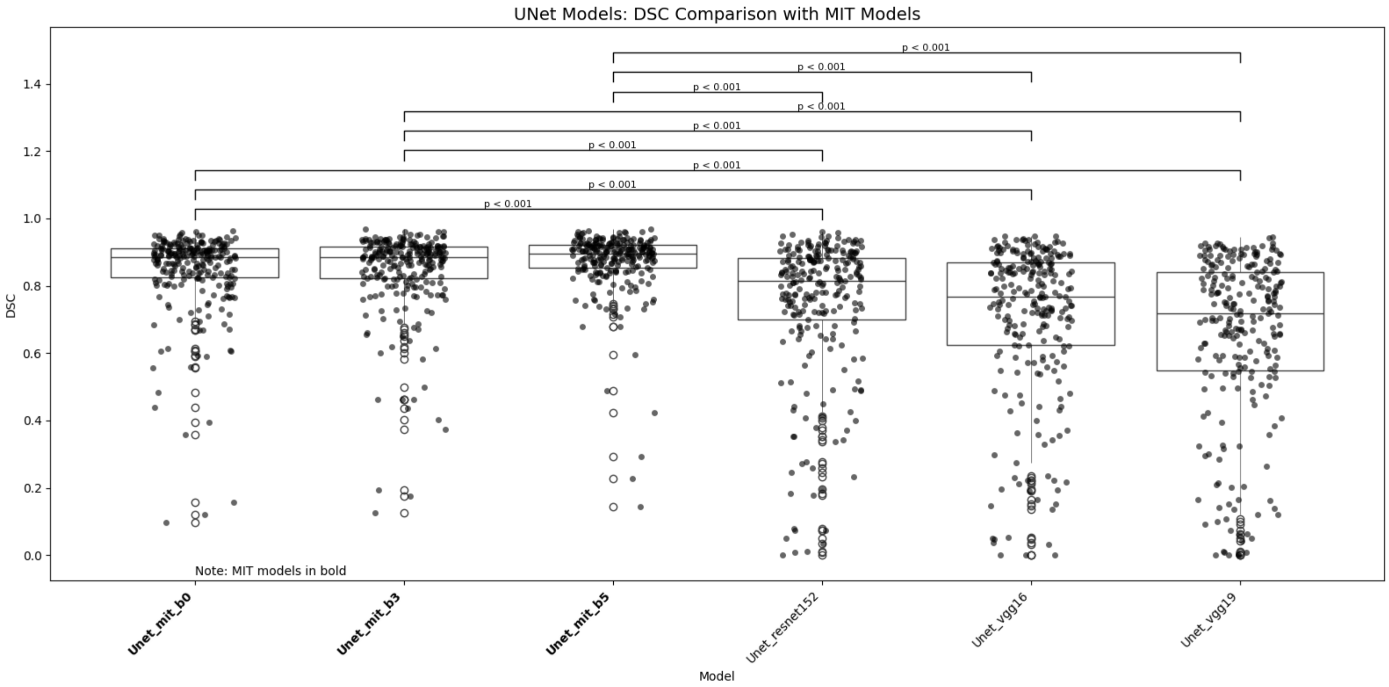

Figure S39. Boxplot of Dice Similarity Coefficient (DSC) distributions for UNet models under high-noise condition for hyperpolarized gas MRI. Under challenging conditions that may arise in clinical hyperpolarized gas MRI, ViT-based models demonstrate the robustness necessary for reliable longitudinal monitoring and treatment assessment.

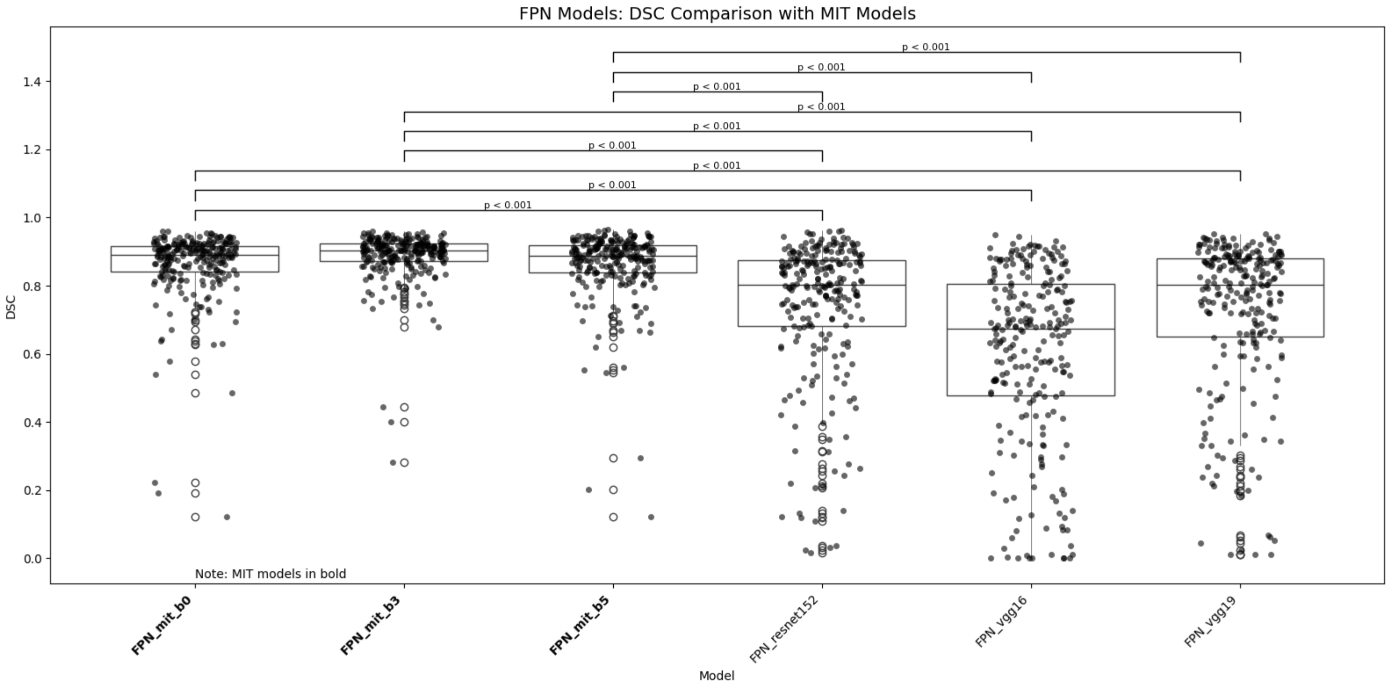

Figure S40. Boxplot of Dice Similarity Coefficient (DSC) distributions for FPN models under high-noise condition for hyperpolarized gas MRI. The superior noise tolerance of transformer architectures is particularly critical in hyperpolarized gas MRI, where segmentation reliability directly impacts clinical metrics such as ventilation defect percentage calculations.

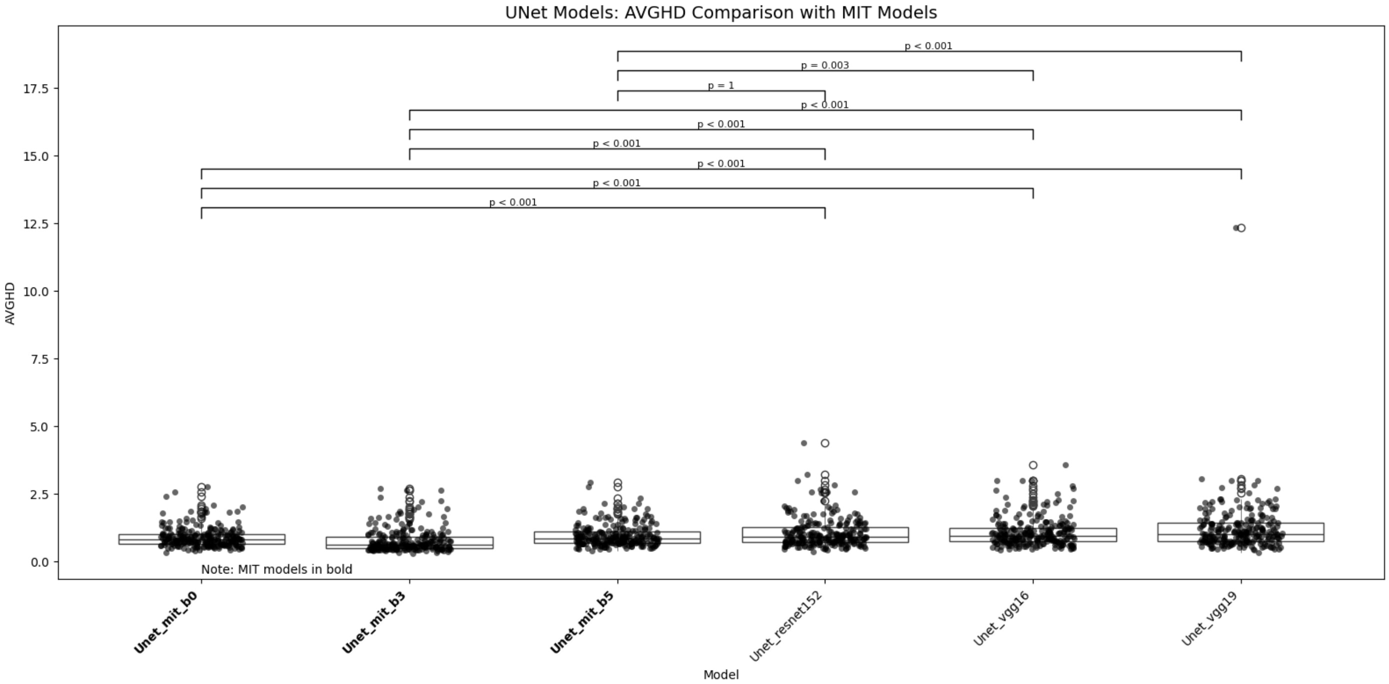

Figure S41. Boxplot of Average Hausdorff Distance (Avg HD) distributions for UNet models under no-noise condition for hyperpolarized gas MRI. Boundary accuracy in hyperpolarized gas MRI is crucial for accurate ventilation mapping, with all models achieving excellent performance under optimal conditions and ViT variants showing slight advantages in consistency.

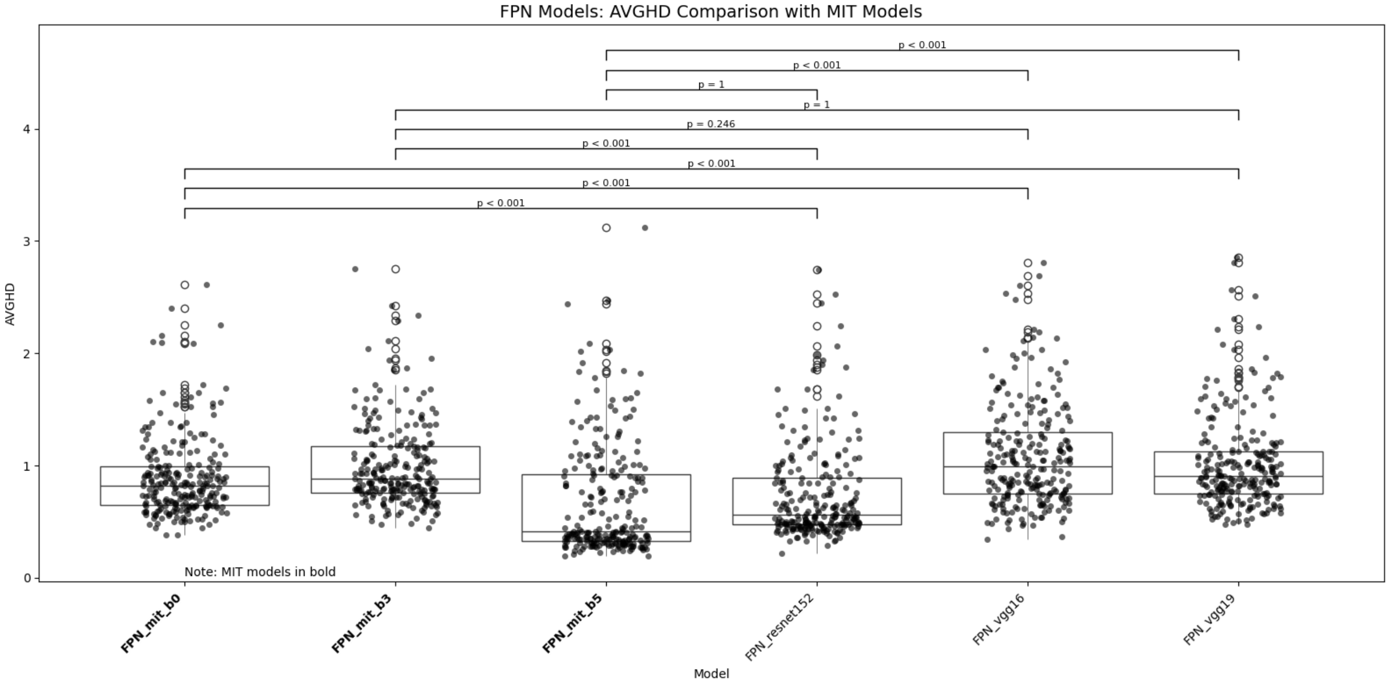

Figure S42. Boxplot of Average Hausdorff Distance (Avg HD) distributions for FPN models under no-noise condition for hyperpolarized gas MRI. FPN architectures demonstrate precise boundary delineation in hyperpolarized gas imaging, with transformer-based models showing marginally superior edge detection that may enhance quantitative analysis accuracy.

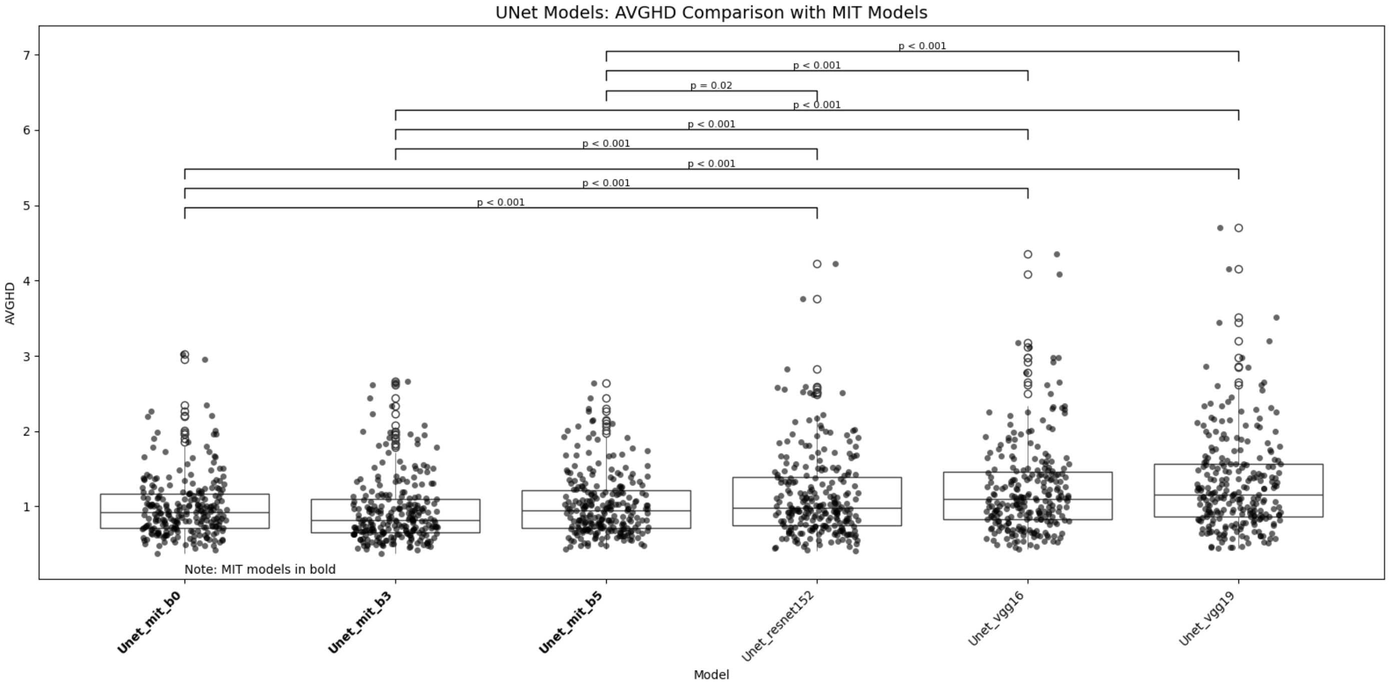

Figure S43. Boxplot of Average Hausdorff Distance (Avg HD) distributions for UNet models under low-noise condition for hyperpolarized gas MRI. The superior boundary preservation of ViT-based models under noise is particularly important in hyperpolarized gas MRI, where accurate segmentation boundaries are essential for ventilation defect identification.

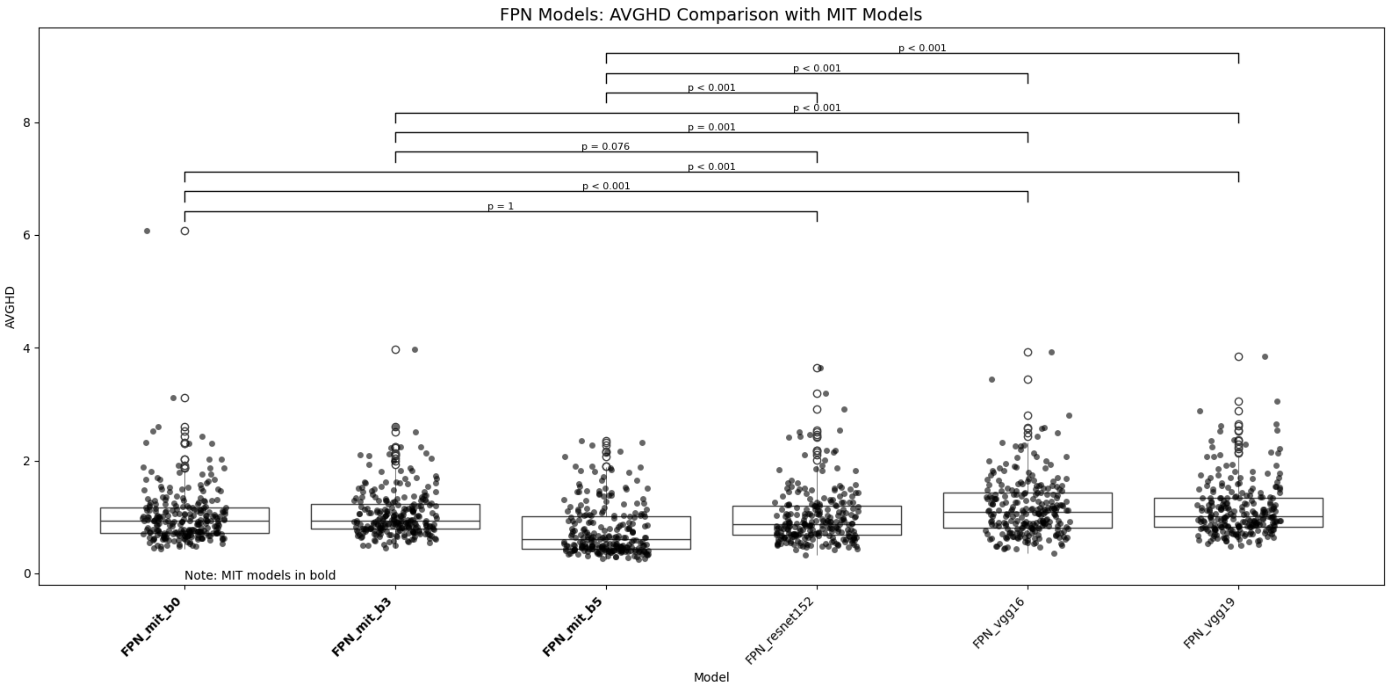

Figure S44. Boxplot of Average Hausdorff Distance (Avg HD) distributions for FPN models under low-noise condition for hyperpolarized gas MRI. Enhanced boundary accuracy of transformer models becomes clinically significant in hyperpolarized gas imaging workflows, where precise lung region delineation impacts downstream quantitative measurements.

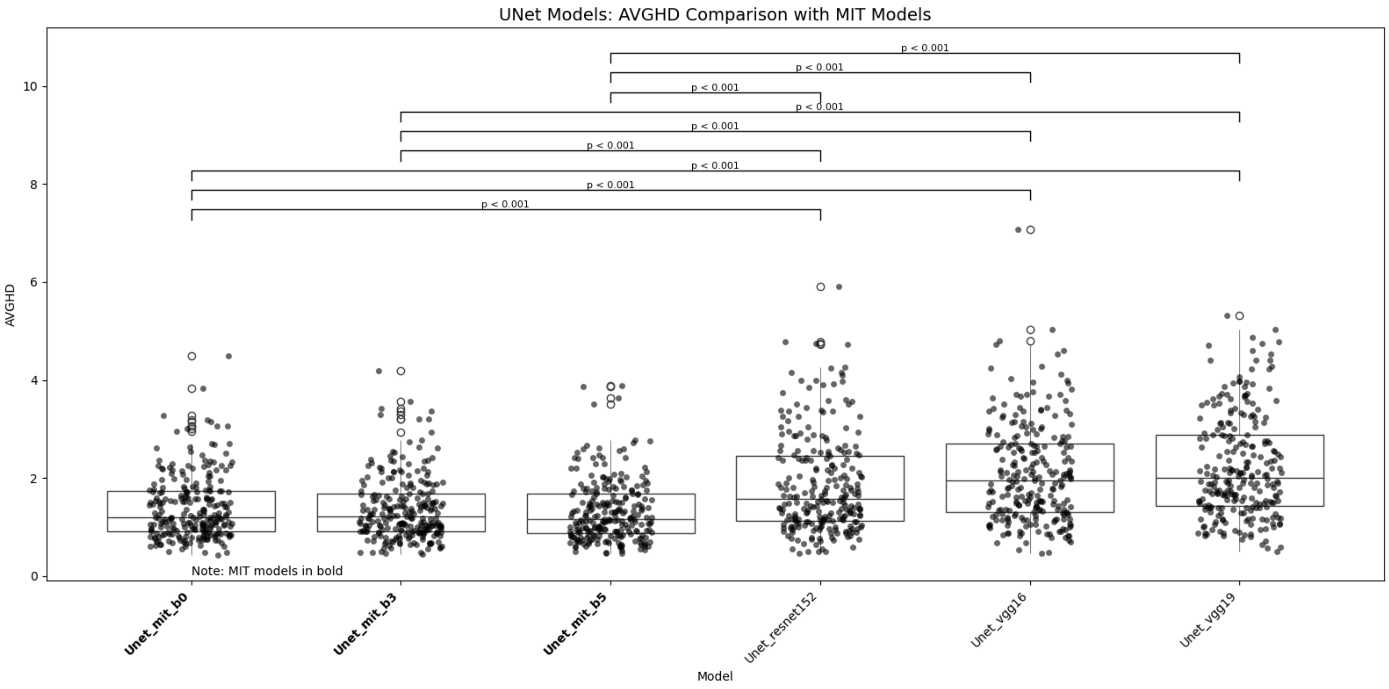

Figure S45. Boxplot of Average Hausdorff Distance (Avg HD) distributions for UNet models under medium-noise condition for hyperpolarized gas MRI. Medium noise levels common in clinical hyperpolarized gas MRI highlight the advantages of ViT architectures in maintaining boundary accuracy necessary for reliable disease monitoring.

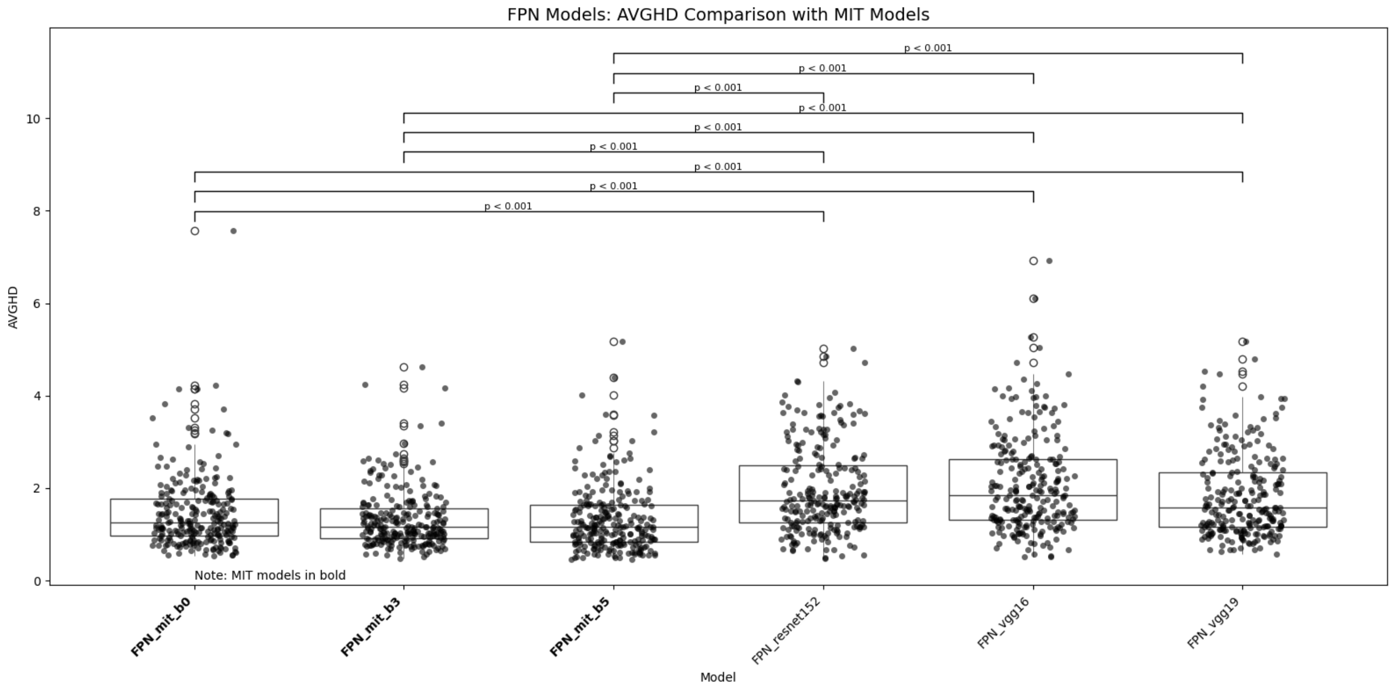

Figure S46. Boxplot of Average Hausdorff Distance (Avg HD) distributions for FPN models under medium-noise condition for hyperpolarized gas MRI. The robust boundary detection of transformer-based FPN models is essential for hyperpolarized gas MRI applications where anatomical precision directly impacts clinical decision-making.

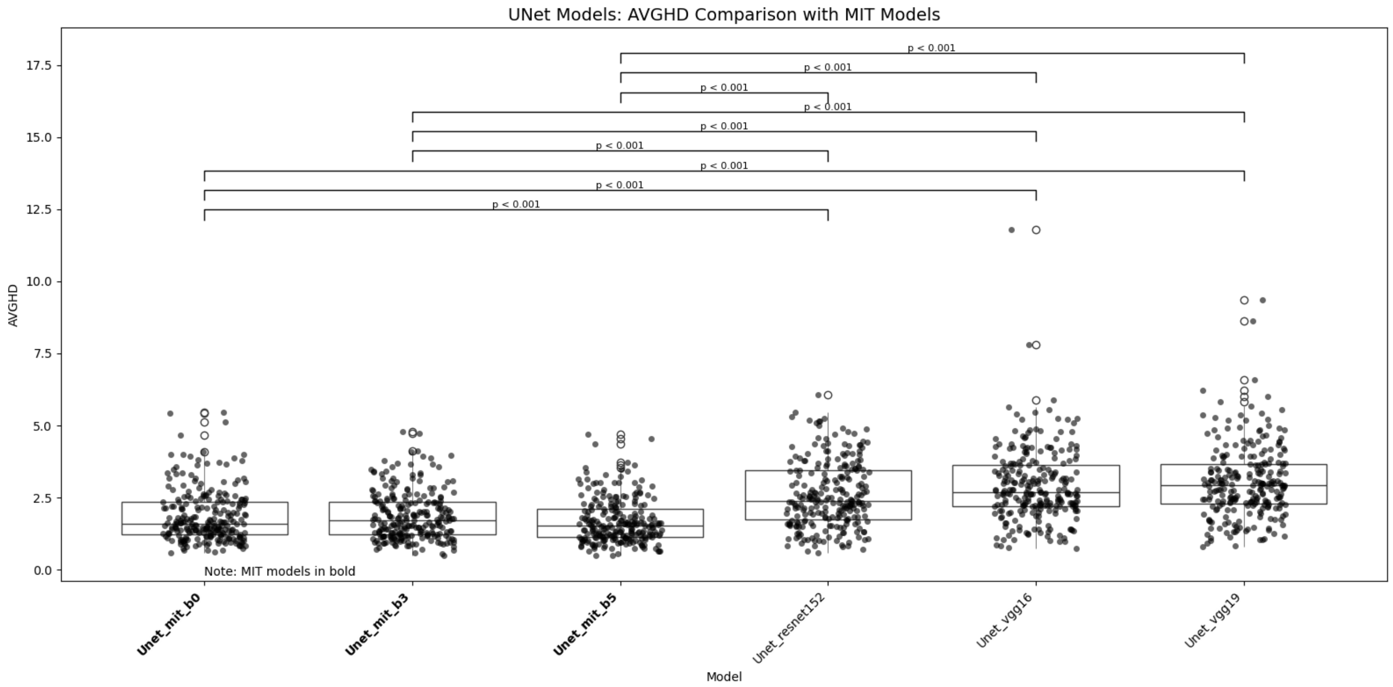

Figure S47. Boxplot of Average Hausdorff Distance (Avg HD) distributions for UNet models under high-noise condition for hyperpolarized gas MRI. Under challenging conditions that may occur in clinical practice, ViT-based models maintain the boundary accuracy essential for confident interpretation of hyperpolarized gas MRI studies.

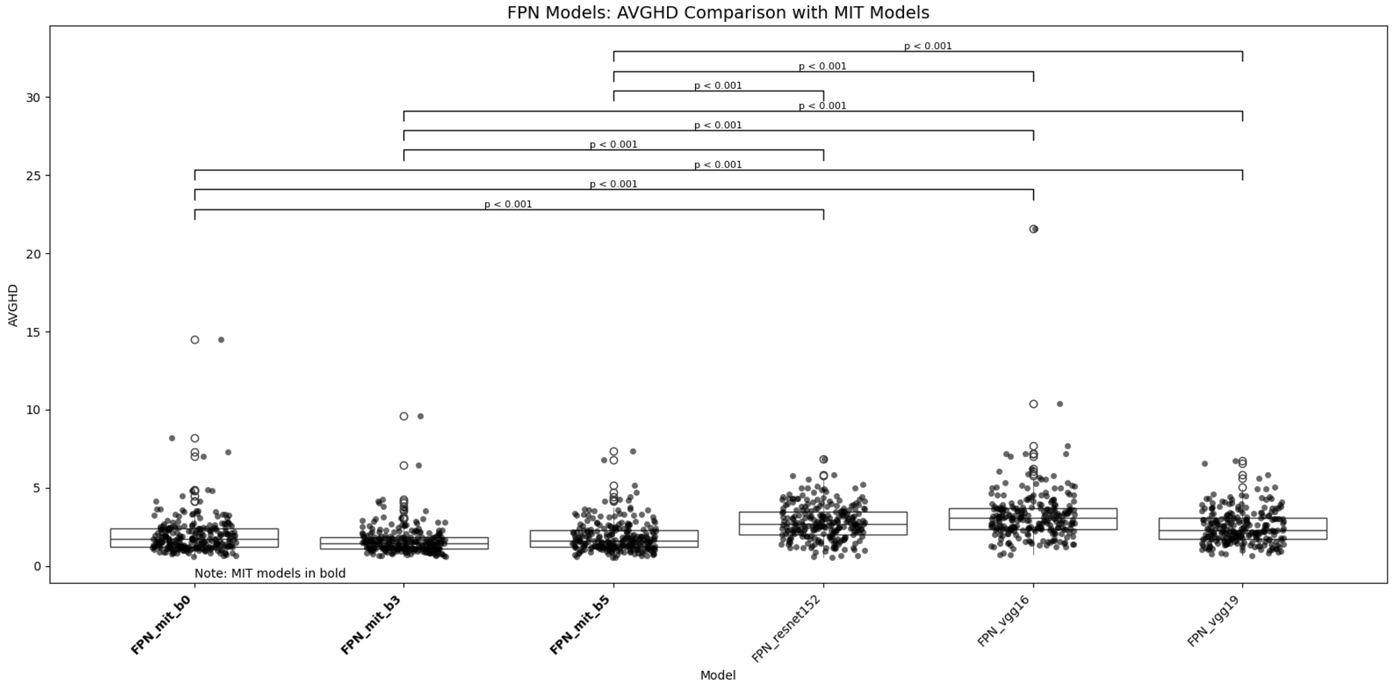

Figure S48. Boxplot of Average Hausdorff Distance (Avg HD) distributions for FPN models under high-noise condition for hyperpolarized gas MRI. The superior boundary preservation of transformer architectures under severe noise conditions ensures reliable segmentation performance in the demanding clinical environment of hyperpolarized gas MRI.

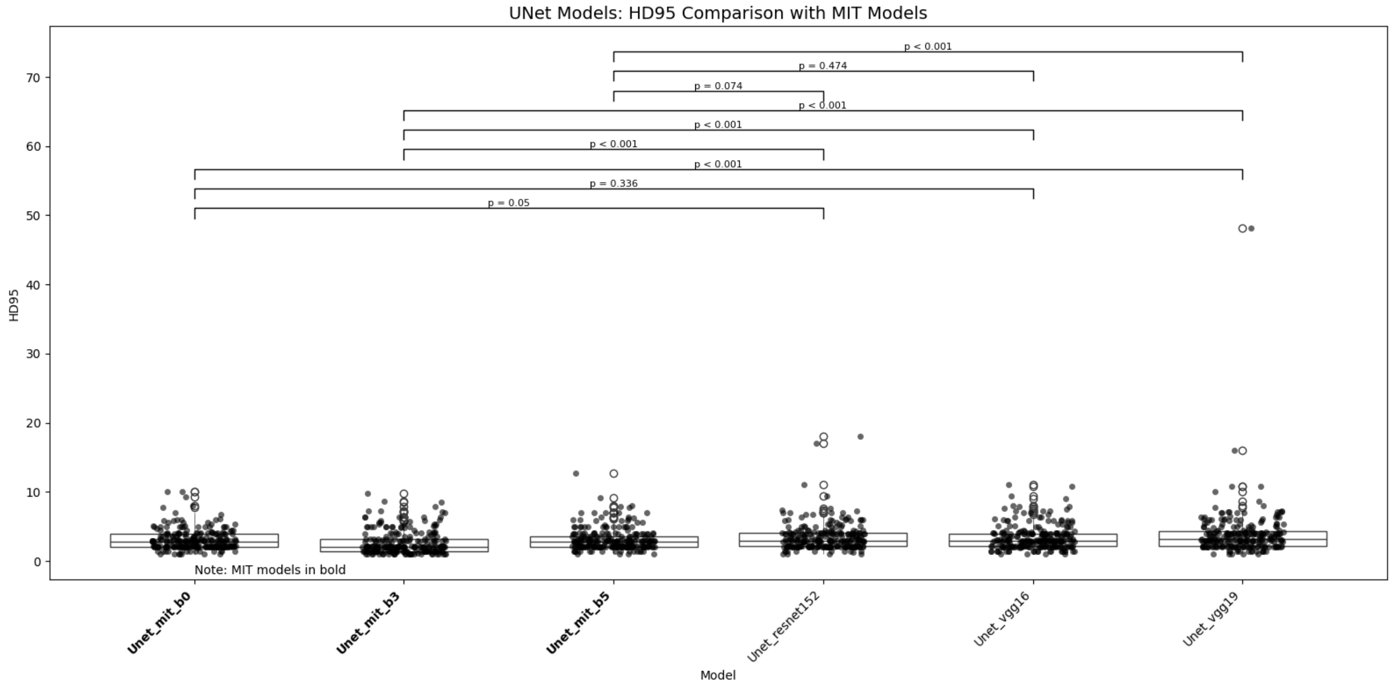

Figure S49. Boxplot of Hausdorff Distance at the 95th percentile (HD95) distributions for UNet models under no-noise condition for hyperpolarized gas MRI. Outlier control in hyperpolarized gas MRI segmentation is crucial for reliable quantitative analysis, with all models achieving low HD95 values under optimal conditions.

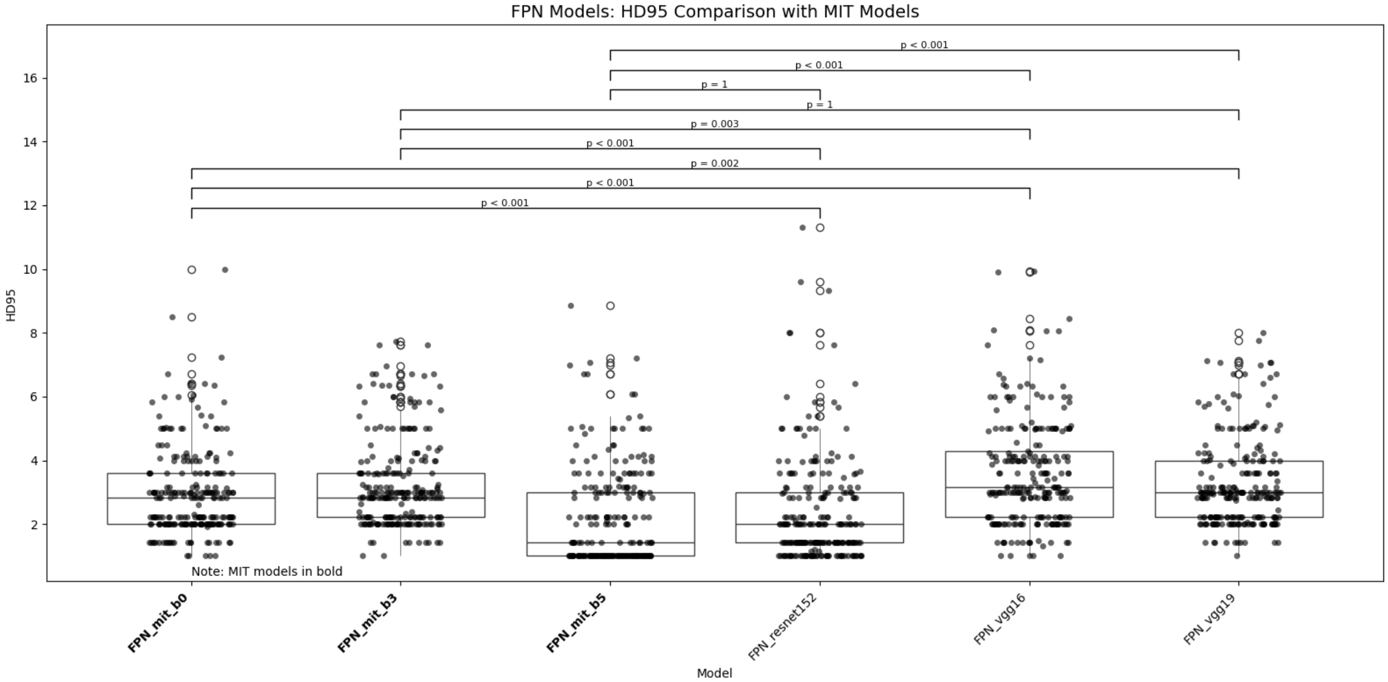

Figure S50. Boxplot of Hausdorff Distance at the 95th percentile (HD95) distributions for FPN models under no-noise condition for hyperpolarized gas MRI. FPN architectures demonstrate excellent control of worst-case boundary errors in hyperpolarized gas imaging, with transformer variants showing slightly superior outlier management.

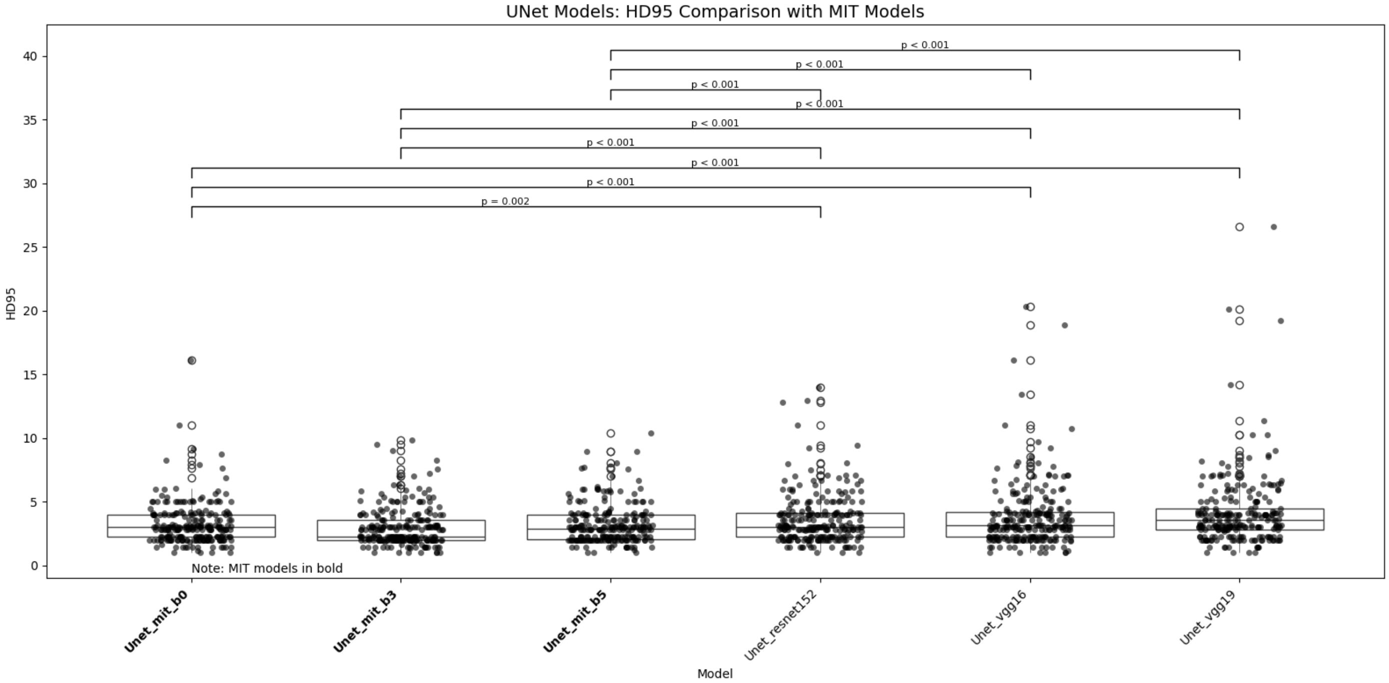

Figure S51. Boxplot of Hausdorff Distance at the 95th percentile (HD95) distributions for UNet models under low-noise condition for hyperpolarized gas MRI. The enhanced outlier control of ViT-based models is particularly valuable in hyperpolarized gas MRI, where extreme segmentation errors can significantly impact clinical metrics.

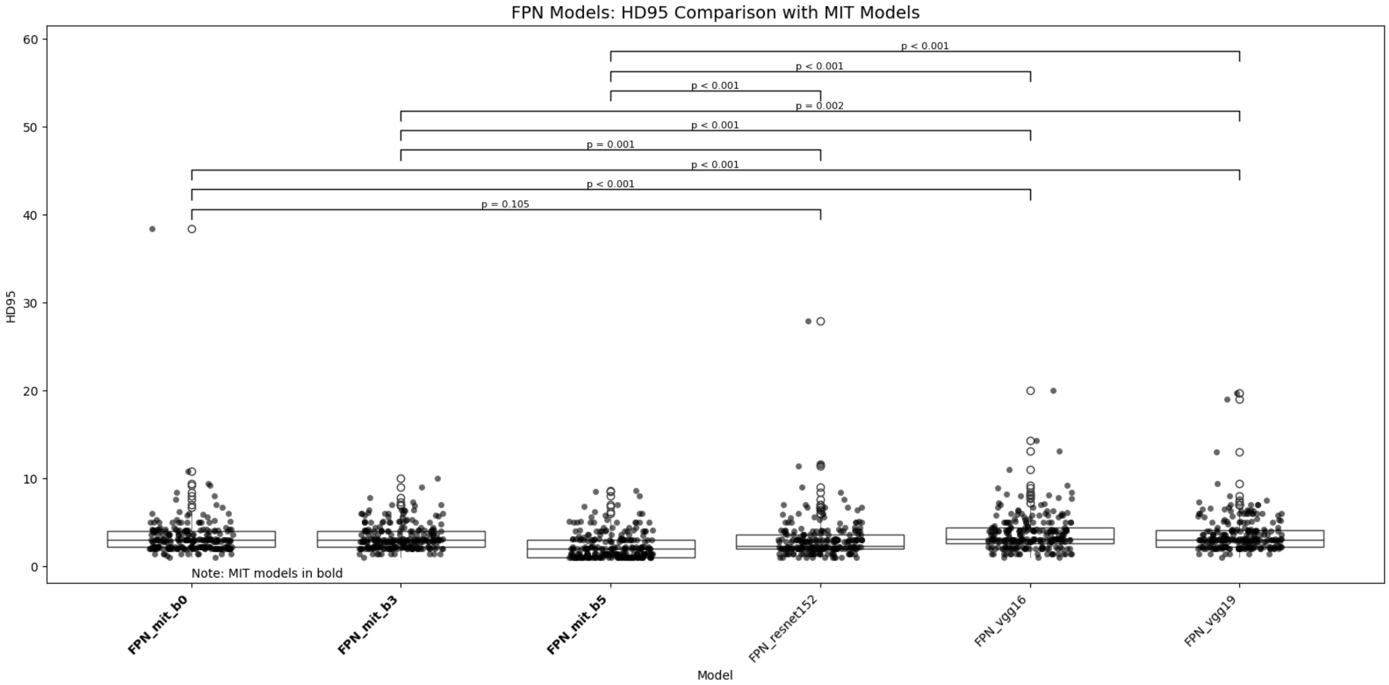

Figure S52. Boxplot of Hausdorff Distance at the 95th percentile (HD95) distributions for FPN models under low-noise condition for hyperpolarized gas MRI. Superior HD95 performance of transformer models ensures more reliable quantitative measurements in hyperpolarized gas MRI applications where precision is paramount.

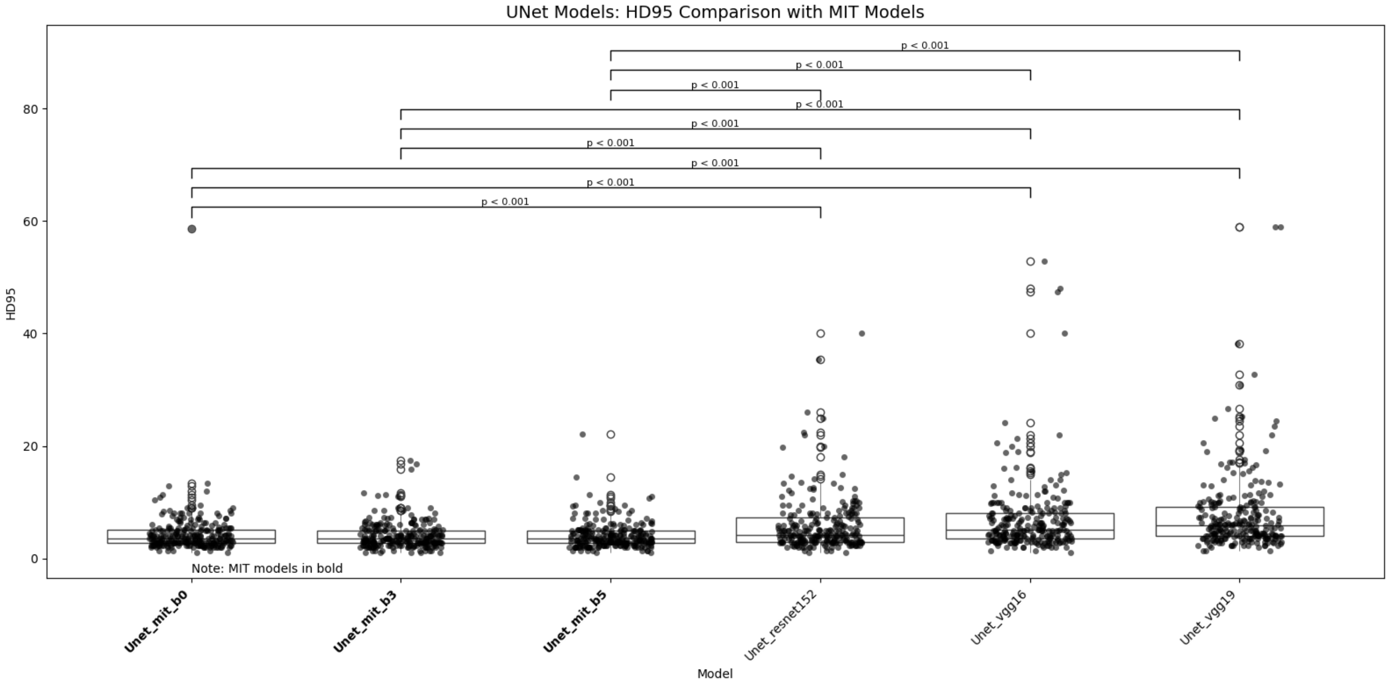

Figure S53. Boxplot of Hausdorff Distance at the 95th percentile (HD95) distributions for UNet models under medium-noise condition for hyperpolarized gas MRI. Under realistic noise conditions in hyperpolarized gas MRI, ViT architectures demonstrate the outlier control necessary for confident clinical interpretation and longitudinal comparison.

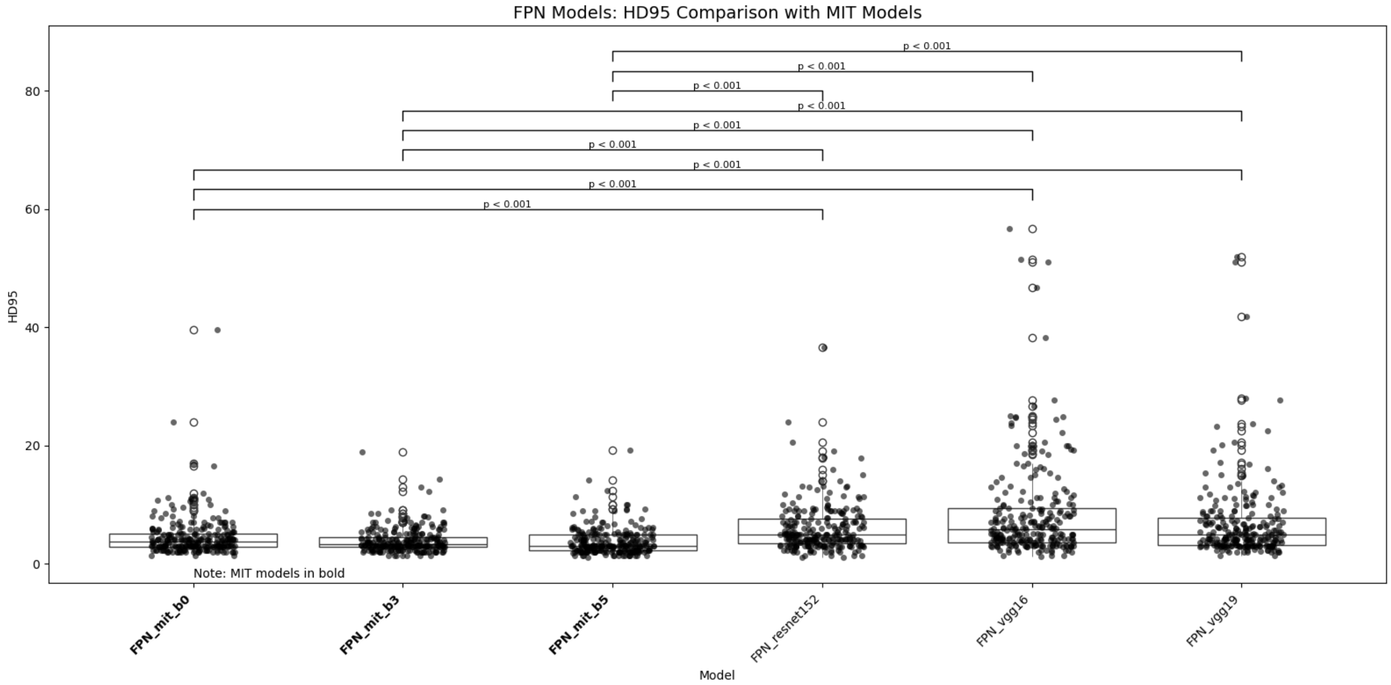

Figure S54. Boxplot of Hausdorff Distance at the 95th percentile (HD95) distributions for FPN models under medium-noise condition for hyperpolarized gas MRI. The robust error control of transformer-based FPN models is essential for maintaining measurement reliability in the challenging imaging environment of hyperpolarized gas MRI.

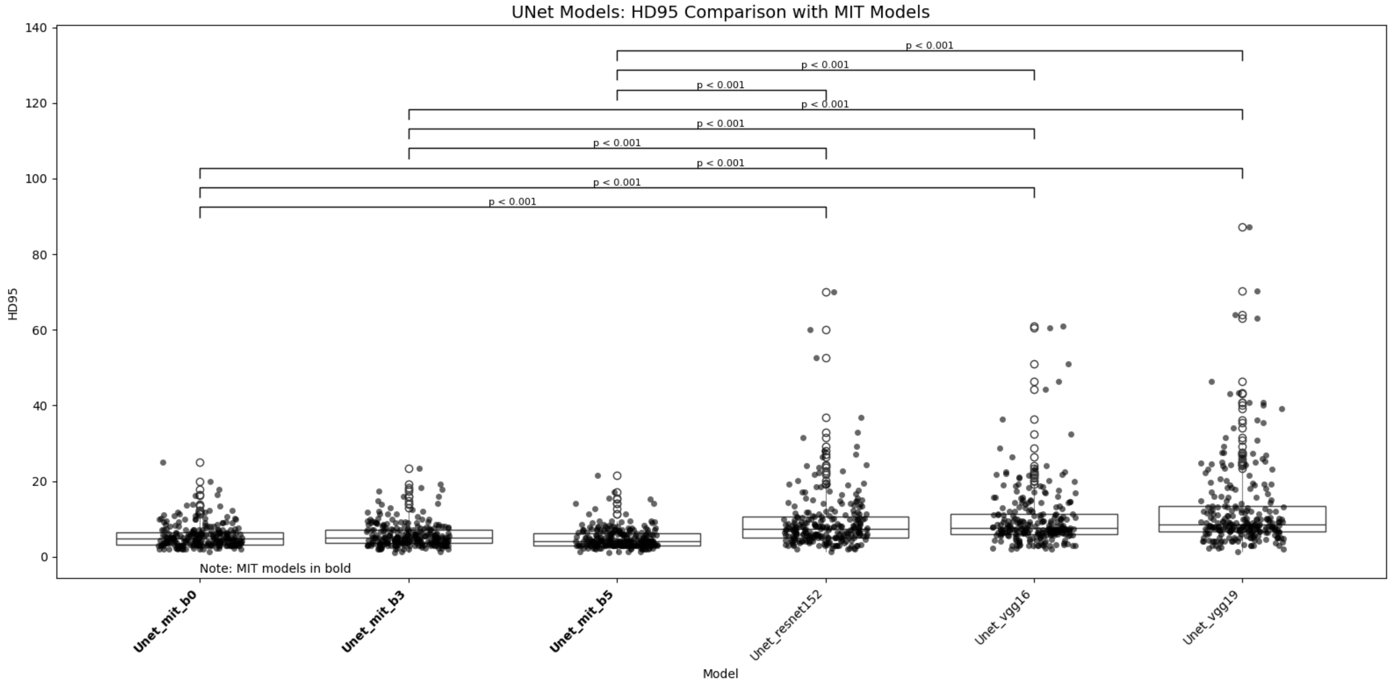

Figure S55. Boxplot of Hausdorff Distance at the 95th percentile (HD95) distributions for UNet models under high-noise condition for hyperpolarized gas MRI. Under severe noise conditions, ViT-based models maintain the outlier control essential for reliable segmentation in hyperpolarized gas MRI clinical workflows.

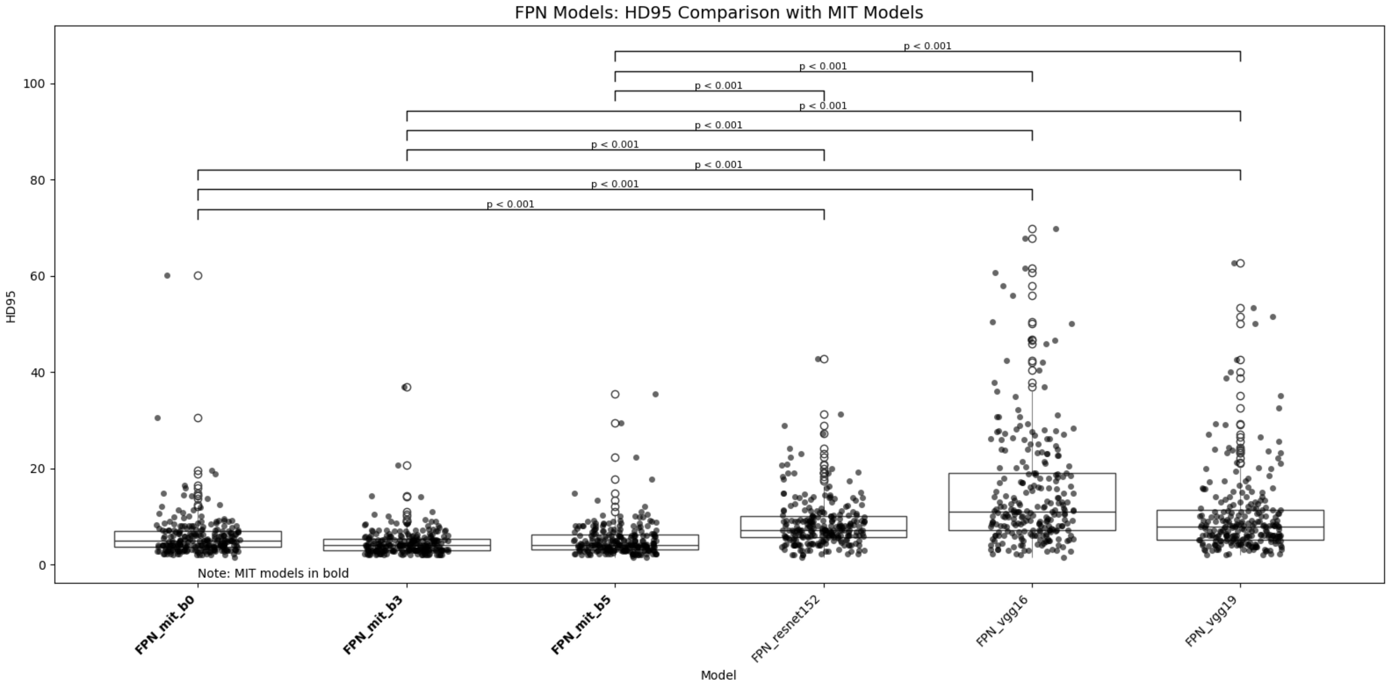

Figure S56. Boxplot of Hausdorff Distance at the 95th percentile (HD95) distributions for FPN models under high-noise condition for hyperpolarized gas MRI. The superior HD95 performance of transformer architectures under challenging conditions ensures robust segmentation reliability in demanding hyperpolarized gas MRI applications.

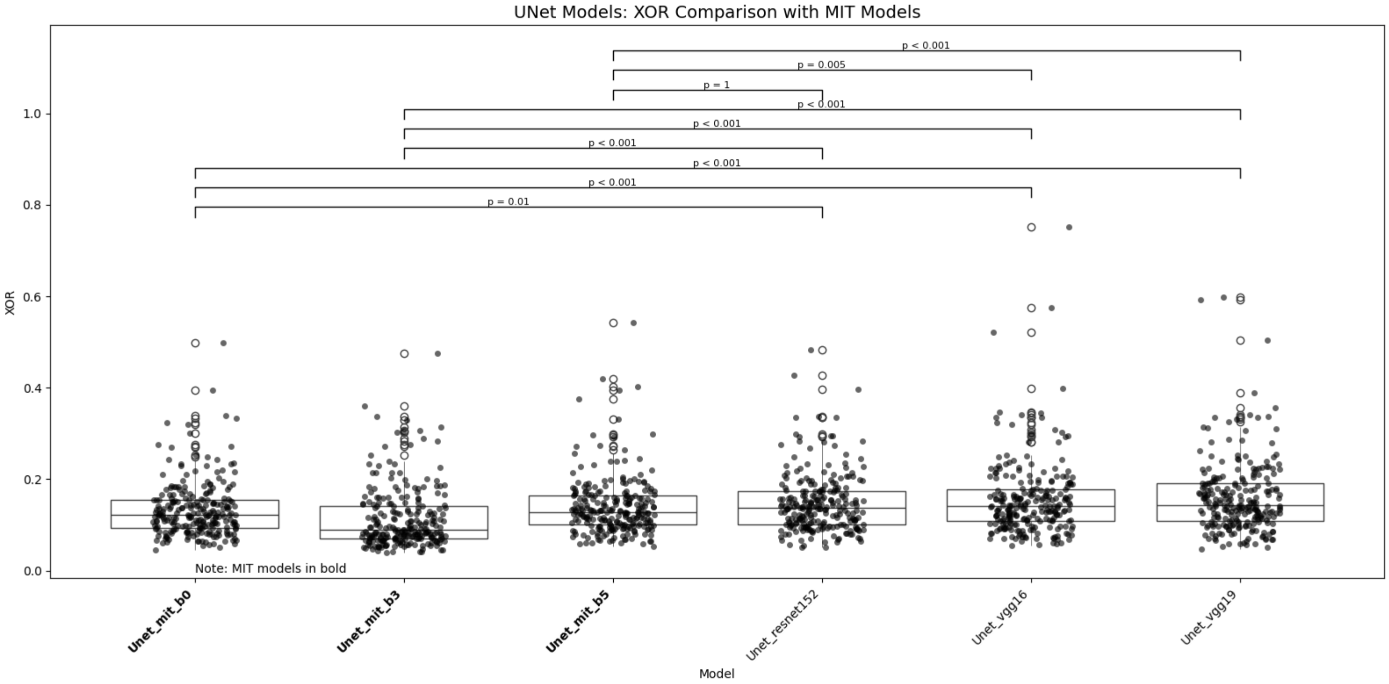

Figure S57. Boxplot of XOR Error distributions for UNet models under no-noise condition for hyperpolarized gas MRI. Pixel-level accuracy is fundamental for quantitative hyperpolarized gas MRI analysis, with all models achieving low XOR error rates under optimal conditions and ViT variants showing enhanced consistency.

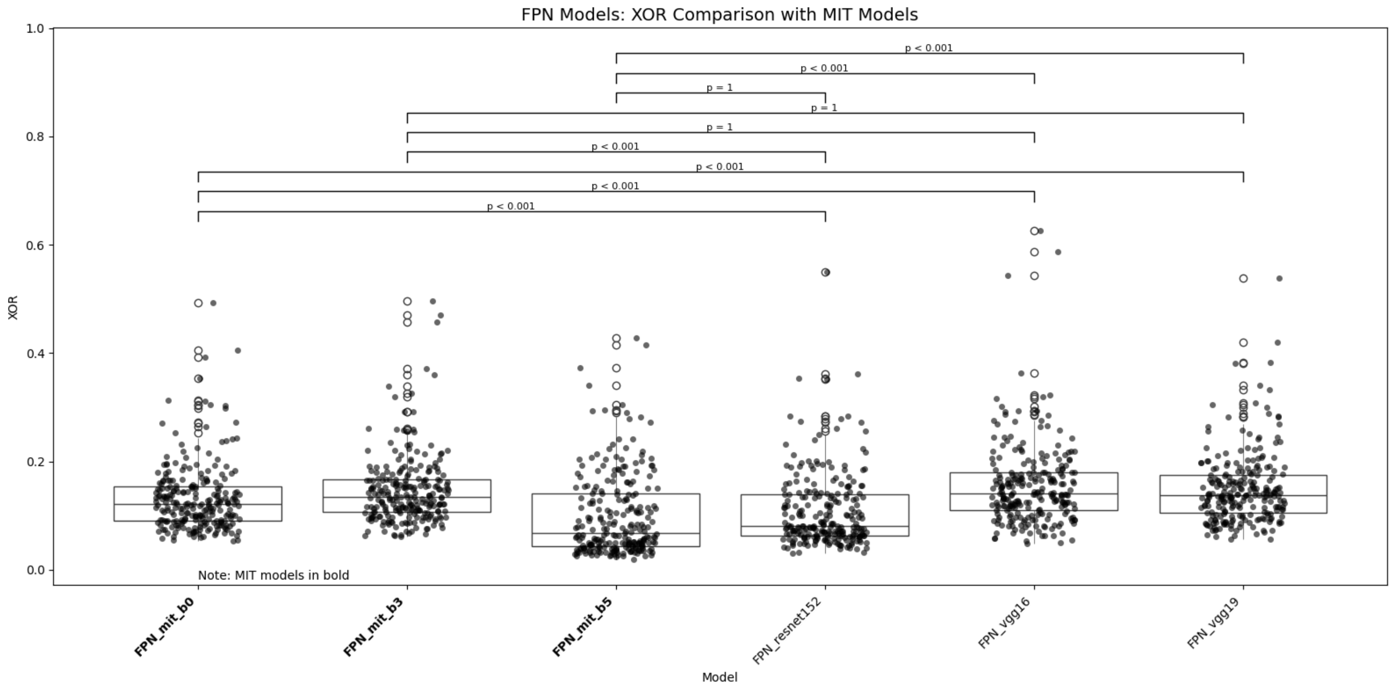

Figure S58. Boxplot of XOR Error distributions for FPN models under no-noise condition for hyperpolarized gas MRI. FPN architectures demonstrate excellent pixel-level precision in hyperpolarized gas imaging, with transformer-based models showing marginally superior accuracy that may benefit detailed ventilation analysis.

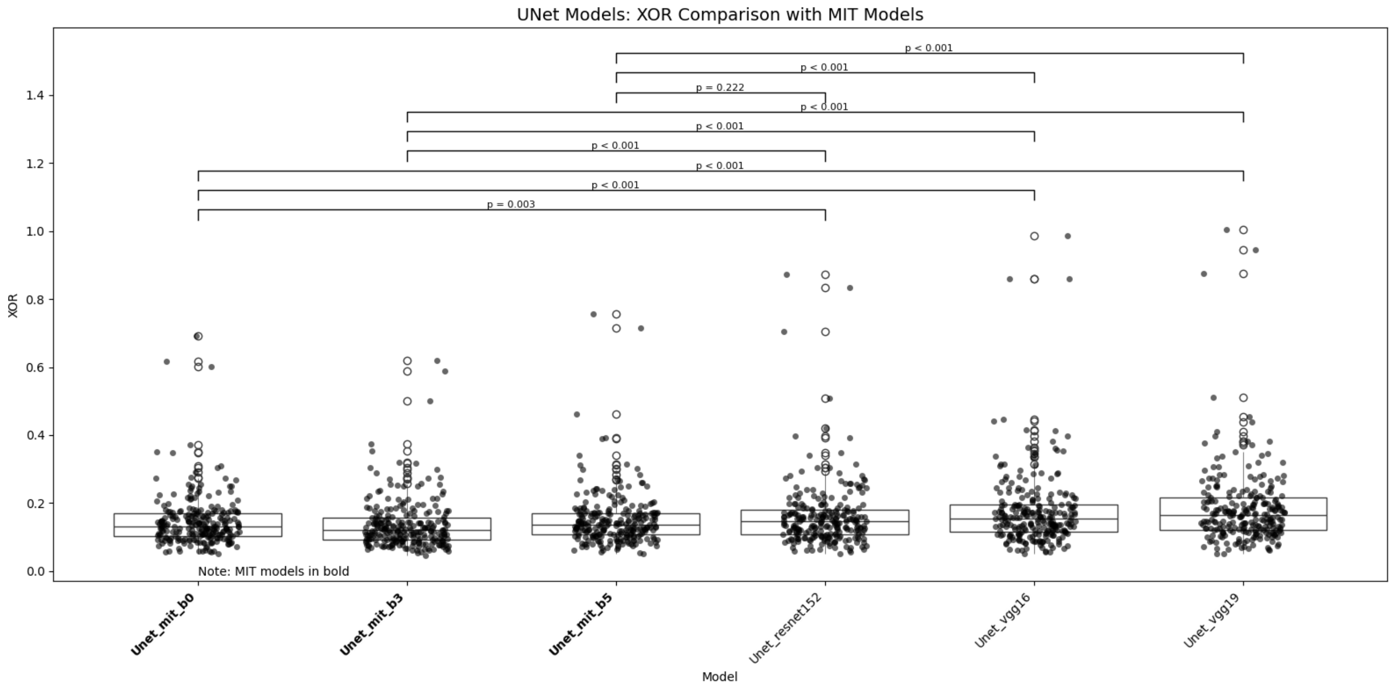

Figure S59. Boxplot of XOR Error distributions for UNet models under low-noise condition for hyperpolarized gas MRI. The maintained pixel-level accuracy of ViT-based models under noise is crucial for hyperpolarized gas MRI applications where precise tissue classification impacts ventilation defect quantification.

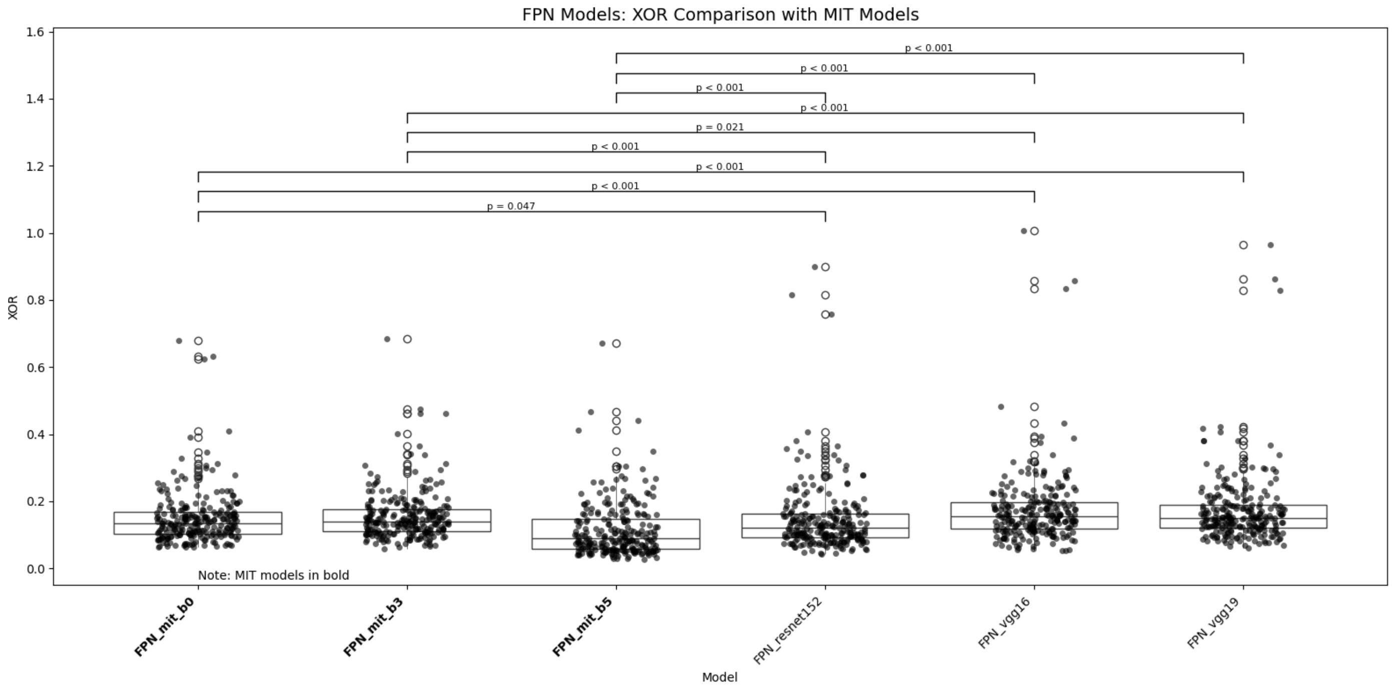

Figure S60. Boxplot of XOR Error distributions for FPN models under low-noise condition for hyperpolarized gas MRI. Enhanced pixel-level precision of transformer models becomes clinically significant in hyperpolarized gas imaging, where accurate segmentation directly affects quantitative biomarker calculation.

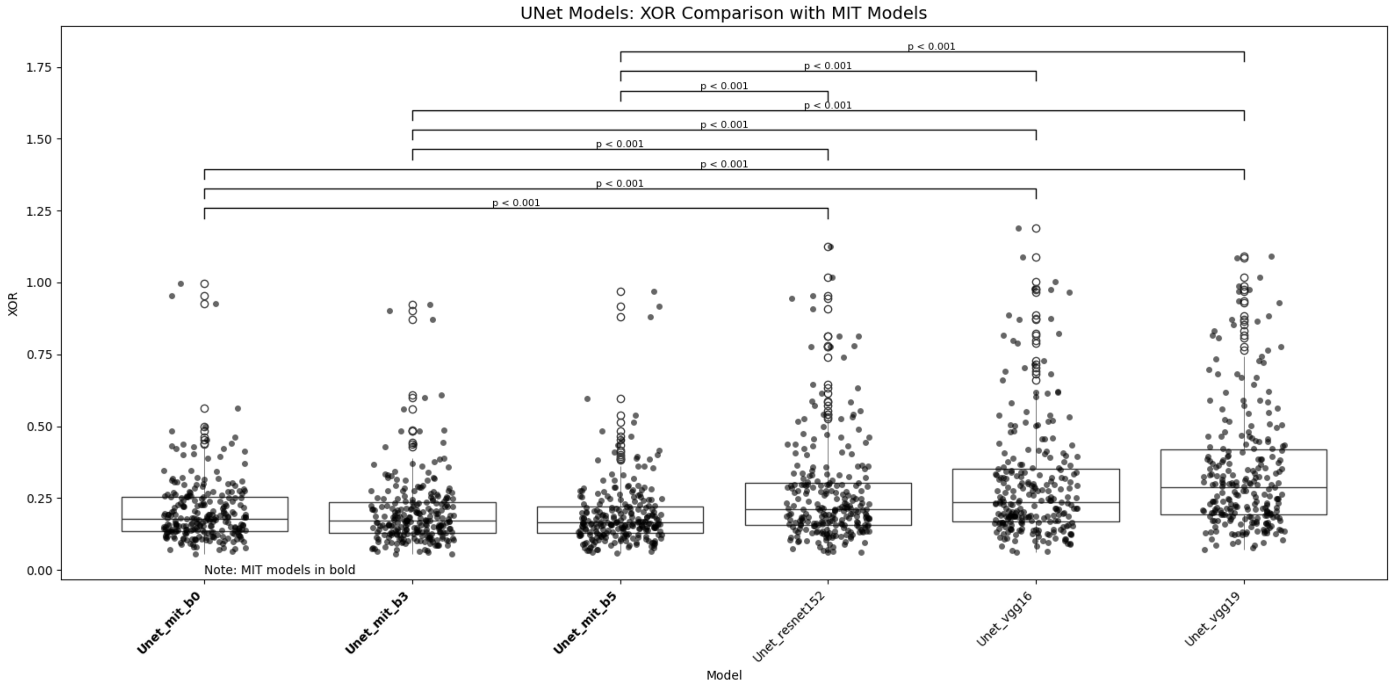

Figure S61. Boxplot of XOR Error distributions for UNet models under medium-noise condition for hyperpolarized gas MRI. Under realistic noise conditions in hyperpolarized gas MRI, ViT architectures maintain the pixel-level accuracy necessary for reliable disease assessment and monitoring.

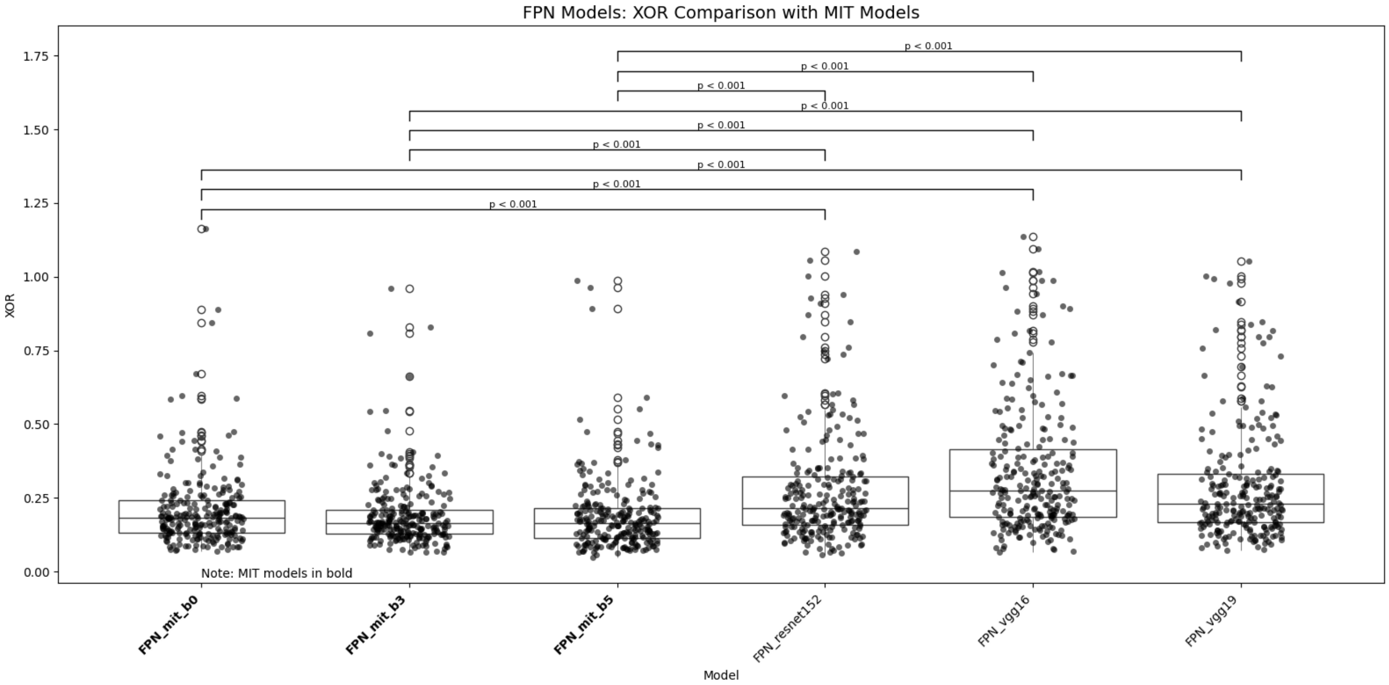

Figure S62. Boxplot of XOR Error distributions for FPN models under medium-noise condition for hyperpolarized gas MRI. The robust pixel-level performance of transformer-based FPN models ensures accurate segmentation in the challenging imaging environment typical of clinical hyperpolarized gas MRI.

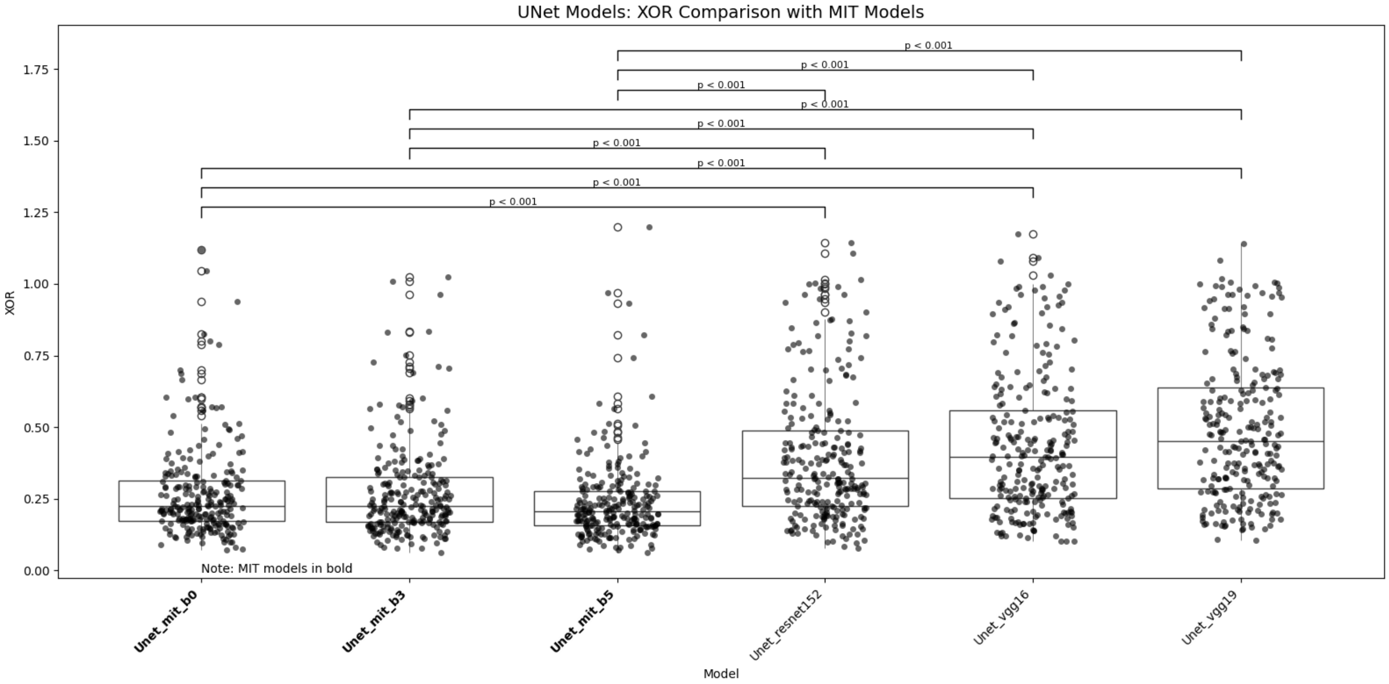

Figure S63. Boxplot of XOR Error distributions for UNet models under high-noise condition for hyperpolarized gas MRI. Under severe noise conditions, ViT-based models demonstrate the pixel-level accuracy essential for confident interpretation and quantitative analysis of hyperpolarized gas MRI studies.

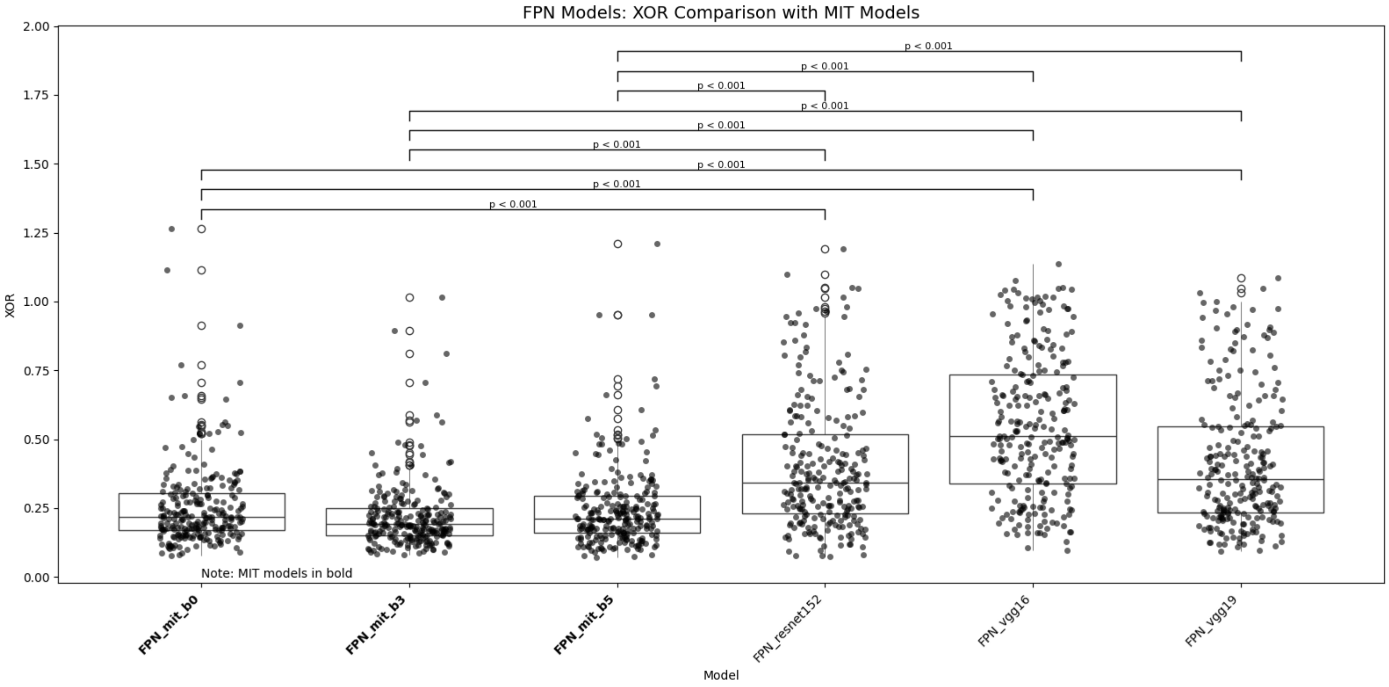

Figure S64. Boxplot of XOR Error distributions for FPN models under high-noise condition for hyperpolarized gas MRI. The superior XOR error control of transformer architectures under challenging conditions ensures reliable segmentation performance critical for clinical hyperpolarized gas MRI applications.

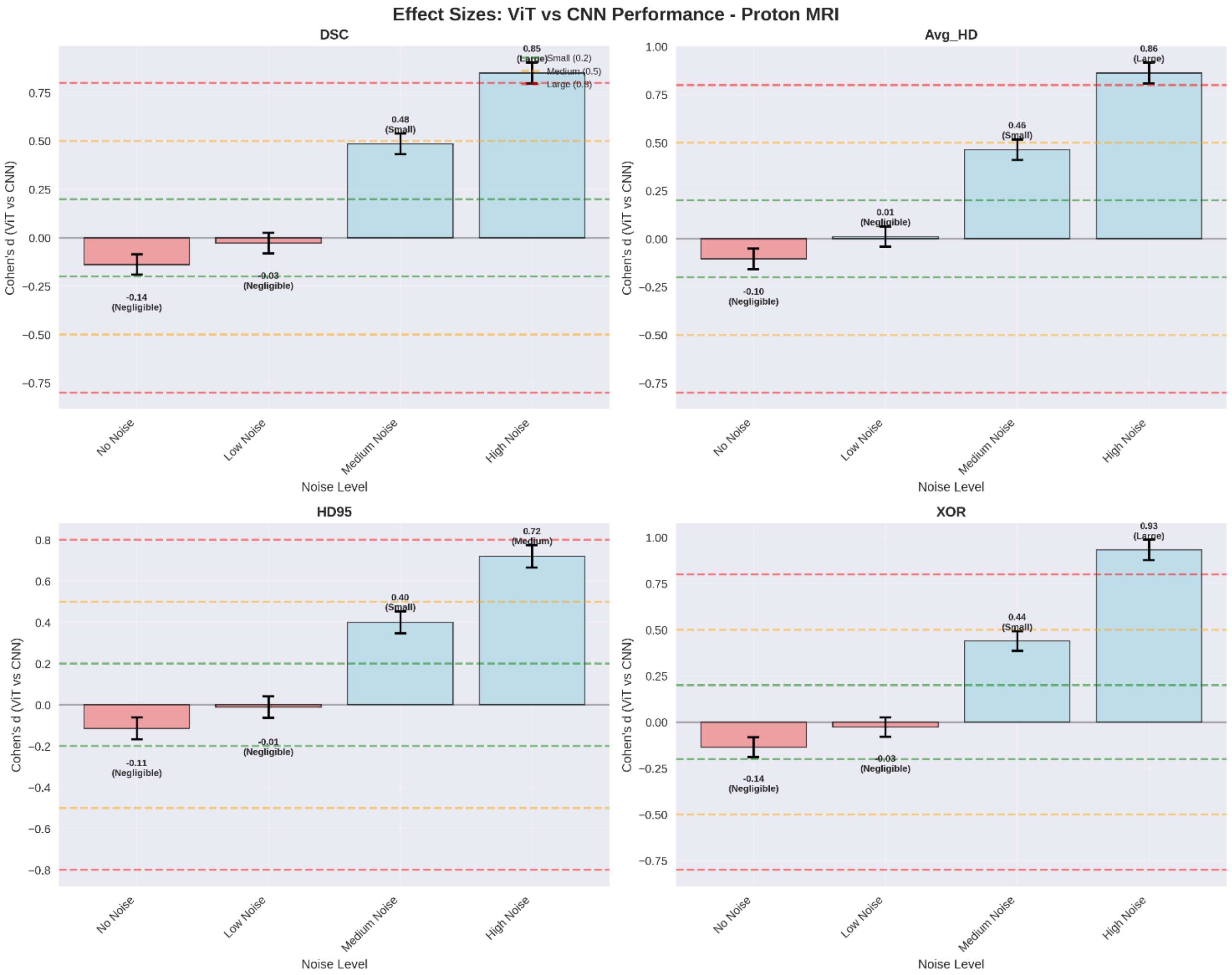

Figure S65. Effect size analysis for ViT versus CNN segmentation performance on proton MRI across noise conditions. Cohen's d values with 95% confidence intervals comparing Vision Transformer (ViT) and Convolutional Neural Network (CNN) models across four evaluation metrics: (A) Dice Similarity Coefficient (DSC), (B) Average Hausdorff Distance (Avg HD), (C) 95th percentile Hausdorff Distance (HD95), and (D) XOR error. Each subplot shows performance across four noise levels: no noise (std = 0.0), low noise (std = 0.05), medium noise (std = 0.15), and high noise (std = 0.25). Positive Cohen's d values indicate ViT superiority, while negative values favor CNN models. Horizontal dashed lines indicate conventional effect size thresholds: small (0.2), medium (0.5), and large (0.8). Error bars represent 95% confidence intervals. Bar colors indicate effect direction (blue = ViT superior, red = CNN superior). Effect size labels show magnitude and interpretation (Negligible, Small, Medium, Large). ViT models demonstrate progressively larger advantages as noise increases, with large effect sizes ($d > 0.8$) observed under high noise conditions across all metrics.

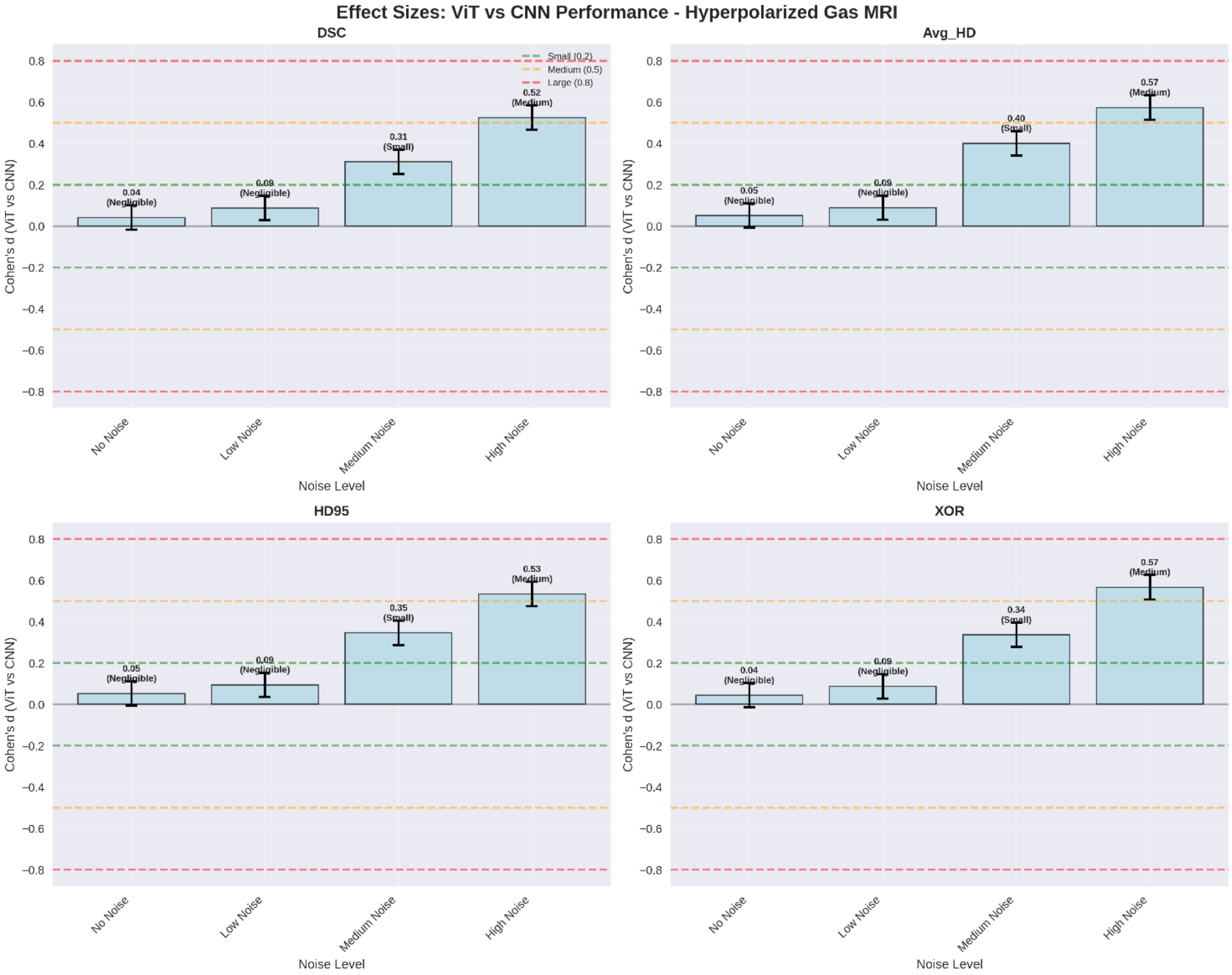

Figure S66. Effect size analysis for ViT versus CNN segmentation performance on hyperpolarized gas MRI across noise conditions. Cohen's d values with 95% confidence intervals comparing Vision Transformer (ViT) and Convolutional Neural Network (CNN) models for hyperpolarized gas MRI segmentation across four evaluation metrics: (A) Dice Similarity Coefficient (DSC), (B) Average Hausdorff Distance (Avg HD), (C) 95th percentile Hausdorff Distance (HD95), and (D) XOR error. Results are shown across four noise levels: no noise (std = 0.0), low noise (std = 0.05), medium noise (std = 0.15), and high noise (std = 0.25). Positive Cohen's d values indicate ViT superiority. Horizontal dashed lines mark conventional effect size thresholds: small (0.2), medium (0.5), and large (0.8). Error bars represent 95% confidence intervals. Bar colors reflect effect direction (blue = ViT superior). Effect size labels indicate magnitude and interpretation. ViT models show consistent advantages that increase with noise level, achieving medium effect sizes (d ≈ 0.5-0.6) under high noise conditions, demonstrating superior robustness in challenging imaging scenarios typical of hyperpolarized gas MRI acquisition.
